# Substrate-derived Sortase A inhibitors: targeting an essential virulence factor of Gram-positive pathogenic bacteria†

**DOI:** 10.1039/d3sc01209c

**Published:** 2023-05-31

**Authors:** Helal Abujubara, Jordi C. J. Hintzen, Shadi Rahimi, Ivan Mijakovic, Daniel Tietze, Alesia A. Tietze

**Affiliations:** a Department of Chemistry and Molecular Biology, Wallenberg Centre for Molecular and Translational Medicine, University of Gothenburg Kemigården 4 412 96 Göteborg Sweden alesia.a.tietze@gu.se; b Division of Systems & Synthetic Biology, Department of Biology and Biological Engineering, Chalmers University of Technology Kemivägen 10 412 96 Göteborg Sweden; c The Novo Nordisk Foundation, Center for Biosustainability, Technical University of Denmark DK-2800 Kongens Lyngby Denmark

## Abstract

The bacterial transpeptidase Sortase A (SrtA) is a surface enzyme of Gram-positive pathogenic bacteria. It has been shown to be an essential virulence factor for the establishment of various bacterial infections, including septic arthritis. However, the development of potent Sortase A inhibitors remains an unmet challenge. Sortase A relies on a five amino acid sorting signal (LPXTG), by which it recognizes its natural target. We report the synthesis of a series of peptidomimetic inhibitors of Sortase A based on the sorting signal, supported by computational binding analysis. By employing a FRET-compatible substrate, our inhibitors were assayed *in vitro*. Among our panel, we identified several promising inhibitors with IC_50_ values below 200 μM, with our strongest inhibitor – LPRDSar – having an IC_50_ of 18.9 μM. Furthermore, it was discovered that three of our compounds show an effect on growth and biofilm inhibition of pathogenic *Staphylococcus aureus*, with the inclusion of a phenyl ring seemingly key to this effect. The most promising compound in our panel, BzLPRDSar, could inhibit biofilm formation at concentrations as low as 32 μg mL^−1^, manifesting it as a potential future drug lead. This could lead to treatments for MRSA infections in clinics and diseases such as septic arthritis, which has been directly linked with SrtA.

## Abstract 2

A series of peptidomimetic Sortase A inhibitors is reported. These compounds show inhibition activity of the Sortase A enzyme and efficiently prevent biofilm formation of *S. aureus*.

## Graphical abstract

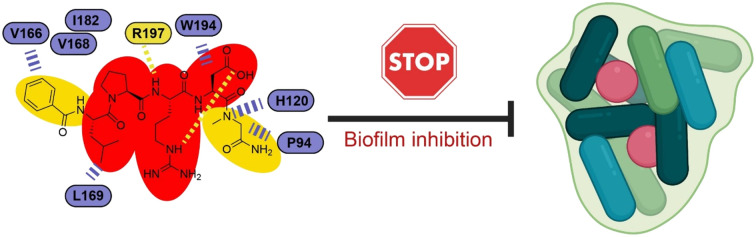

## Introduction

The emergence of bacterial resistance to conventional antibiotics has become a global problem with a considerable impact on the clinical efficacy of currently used treatments. The mechanisms of these conventional antibiotics are mainly associated with the prevention of bacterial growth (bacteriostatic) and inducing bacterial cell death (bactericidal), which is typically achieved by inhibiting vital bacterial metabolism such as nucleic acid, protein and cell wall synthesis.^1^ In these approaches a high selective pressure is exerted on the bacterium, by threatening its viability, leading to a high pressure for the bacteria to develop resistance against these drugs.^1^ Moreover, antibiotic resistance can arise as a result of genetic mutations or through the exchange of resistance genes between bacteria.^2^ Recently, antivirulence agents have shown promising features in treating resistant bacterial infections and avoiding bacterial resistance.^3–5^

Bacterial virulence factors are molecules that enable bacteria to colonize and infect the host, as well as increase their capacity to damage the host tissues.^6^ Different types of virulence factors have been identified, such as surface proteins, toxins, hydrolytic enzymes and capsules.^6,7^ Targeting these virulence factors, instead of conventional targets of antibiotics, has gained increasing interest as these treatments have no bactericidal activity and no effect on bacterial cell growth, but interfere mainly with bacterial mechanisms that initiate the infection.^4^ In fact, antivirulence treatments disarm pathogenic bacteria rather than killing them and, as a result, employ low selective pressure to induce the development of antibiotic resistance.^3,8,9^

Bacterial adhesion to the host tissues is the first crucial step for infection and colonization,^10^ playing a critical role in the formation of biofilms which protect the pathogen from the host immune system.^11^ Inhibition of the bacterial adhesion process has been recognized as a promising anti-virulence approach.^12^ Pili in Gram-positive bacteria are structural protein motifs that are covalently linked to the peptidoglycan layer.^13^ These cell wall anchored (CWA) proteins play a major role in adhesion since they are used to target the host's extracellular matrix proteins such as collagen, fibrinogen and fibronectin. A subfamily of CWAs, the MSCRAMMs (microbial surface components recognizing adhesive matrix molecules), are covalently linked to their cell wall peptidoglycans by unique cysteine transpeptidases called sortases.^13^ Sortase enzymes are ubiquitous in Gram-positive bacteria and have an important role in bacterial virulence. Among these, Sortase A (SrtA) is considered a housekeeping sortase and is of great interest as a potential target for antivirulence treatments.^14^

**Fig. 1:**
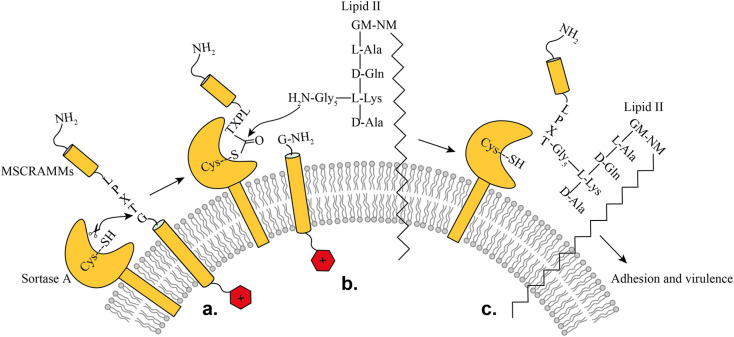
Representation of the SrtA catalyzed surface protein anchoring mechanism: (a) recognition of target MSCRAMMs, (b) transthioesterification, (c) transpeptidation.

SrtA is a membrane-bound transpeptidase consisting of an N-terminal transmembrane region and a C-terminal catalytic region.^14^ SrtA recognizes MSCRAMMs by their sorting signal consisting of an LPXTG motif, where X represents any amino acid.^14^ The anchoring process is initiated by recognition of the LPXTG motif, followed by a transthioesterification reaction, which cleaves the peptide bond between the threonine and the glycine residue of the sorting motif, resulting in the formation of a thioester acyl-enzyme intermediate (Fig. 1). Subsequently, a second transpeptidation reaction mediated by SrtA is performed between the thioester intermediate and the pentaglycine (Gly5) unit of the cell wall molecule lipid-II, leading to the product being covalently anchored to the cell wall where it can enable adherence of the bacteria to the host cells and tissue (Fig. 1).^10^

Structurally, sortase enzymes consist of an eight-stranded β-barrel fold, connected by random coil loops.^14^ The active site of the enzyme contains a highly conserved catalytic triad consisting of His120, Cys184 and Arg197 at the end of a large groove along one side of the β-barrel. The loops β2/H1, β4/H2, β6/β7 and β7/β8 form the wall of the groove, while strands β4 and β7 constitute the floor of the groove. It was revealed that the enzyme recognizes the LPXTG sorting signal through the large groove that leads into the active site,^15^ which was recently confirmed through the first solved X-ray structures of SrtA bound to its substrate peptides.^16^ The β6/β7 loop was found to be highly mobile in the absence of a ligand and plays an important role in catalysis. Upon ligand binding, the β6/β7 loop transits from a structurally disordered and open conformation to an ordered closed conformation containing a 3_10_ helix.^15^ This conformational change pushes the substrate deeper into the groove of the active site and initiates catalysis.

Inhibition of SrtA has been shown to cause a reduction of biofilm formation in some *Staphylococcus aureus* strains and loss of binding activity to fibronectin, fibrinogen and immunoglobin G, leading to a reduction of bacterial virulence.^17^ Other than this, SrtA possesses several features making it a prominent target for prevention of bacterial virulence. Firstly, SrtA is not essential for bacterial survival and growth, which is an essential characteristic of antivirulent targets. Importantly, no homologues of SrtA exist in humans, which means that selective inhibition should be possible. Finally, because SrtA is a membrane associated protein, it is a relatively easy target, as there is no requirement for the inhibitors to cross the bacterial outer membrane and reach the cytoplasm.

Several diseases have been directly linked with the activity of SrtA so that inhibition of this enzyme provides potential treatment options against these diseases, making it a prime target for drug development. Over 25 years ago it was already identified that typical hospital bacteria such as *S. aureus*, *Staphylococcus epidermidis*, and *Enterococcus faecalis* were resistant to most known treatments at this time.^18^ Particularly methicillin-resistant *S. aureus* (MRSA) infections pose a huge threat to modern-day clinics, with high reported morbidity and mortality rates.^19^ Therefore, novel treatments for these infections are in high demand, and inhibition of SrtA could serve as a long-term solution, due to its antivirulence activity. Septic arthritis, a severe infection of synovial fluid and joint tissues with nearly 50 000 annual cases in Western Europe and the US caused by different bacteria, viruses or fungi, has been directly linked with the activity of *S. aureus* SrtA.^20–23^ It was shown that SrtA-knockout mice infected with *S. aureus* strains did not show severe symptoms of septic arthritis, with increased survival rates and significantly lower weight loss reported. Interestingly, knockout of the closely related Sortase B did not show such an effect, pinpointing SrtA as an essential factor leading to the advent of this disease.^24^ Septic arthritis is particularly prevalent in children and treatments commonly rely on conventional antibiotics, with an often complicated recovery.^25^ Therefore, specific and effective treatments are highly desired, of which SrtA inhibition is a prime candidate.

To date, several SrtA inhibitors have been identified, derived from different types of natural products, small organic molecules and peptides.^5^ Among the peptide-based inhibitors, only modest inhibition of SrtA efficacy was reported to date.^4^ Most compounds are based on the LPXTG sorting signal and replaced the Thr–Gly scissile bond with a variety of functional groups. Reversible inhibitors containing a phosphonic ester bond were shown to have poor inhibition.^26^ Several potential covalent inhibitors were also developed, containing chloromethyl ketone, diazo ketone, vinyl sulfones, sulfhydryl, cyanoalkene groups or a thiol-containing threonine analog, however, none of these peptides displayed exceptional inhibitory potential.^27–30^ Finally, a virtual screening-based approach was employed to identify the strongest peptidomimetic SrtA inhibitor to date.^31^ The peptide PEG_2000_-LPRDA-NH_2_ was reported to have an IC_50_ of 10.61 μM and showed inhibition of biofilm formation by interaction with fibronectin and by invasion into the host tissues in *S. aureus* treated with the oligopeptide.^31^

Still, none of the previously published compounds are currently used clinically and among the peptidomimetic inhibitors, only high micromolar inhibition has been achieved. Furthermore, most of the tested molecules are covalent inhibitors, which are prone to have multiple off-target effects with other cysteine proteases in the body. Therefore, we set out to systematically develop potentially potent peptide-based antivirulence compounds as SrtA inhibitors.

## Results and discussion

### The initial design of peptidomimetic compounds – the first generation of inhibitors

**Fig. 2:**
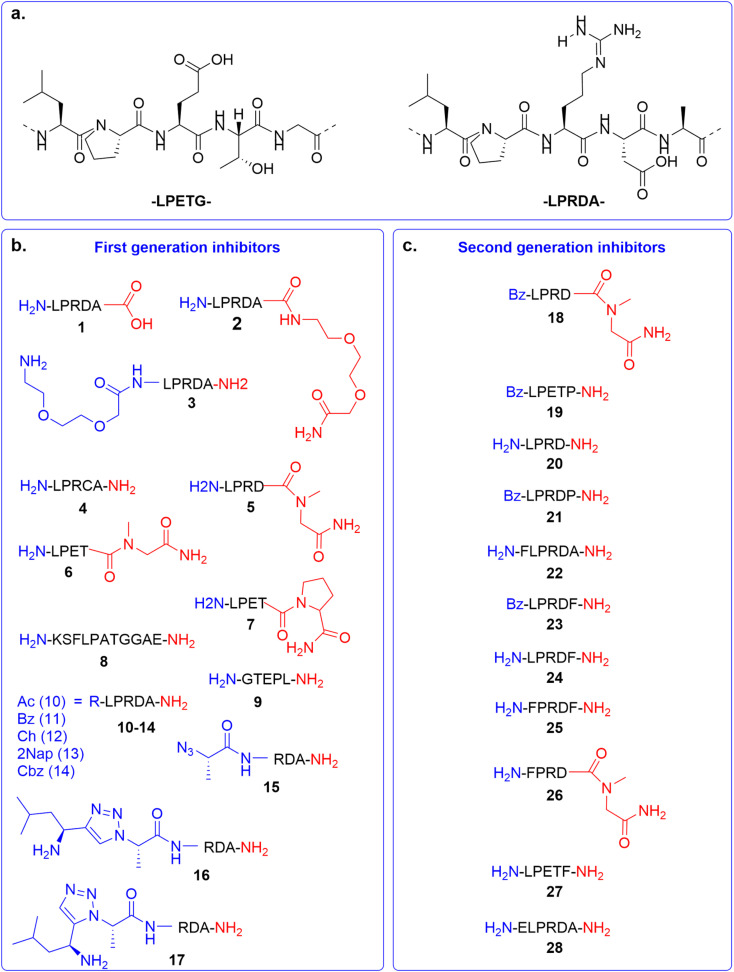
Overview of SrtA inhibitors 1–28 studied in this work, modifications at the C-terminal are colored in red and at the N-terminal in blue. (a) The SrtA recognition motif^32^ and a previously identified peptidomimetic inhibitor^31^ used as the basis for our inhibitor design. (b) First generation of inhibitors; (c) second generation of inhibitors.

In the first generation of compounds, four series of peptidomimetic SrtA inhibitors were developed. The structure of the first series of peptidomimetic inhibitors (1–5) was inspired by the PEG_2000_-LPRDA-NH_2_ structure, reported as a SrtA inhibitor by Wang *et al.* with an IC_50_ of 10.61 μM (Fig. 2a).^31^ In order to investigate the impact of the PEG_2000_ N-terminal modification on LPRDA regarding the inhibitory activity, peptide 1 was included to study the lack of the PEG_2000_ unit. Additionally, for peptides 2 and 3 an amino-3,6-dioxaoctanoic acid (Ado) group, which is composed of two PEG units, was added to either the C- or N-terminus. Compound 4, containing the D4C substitution, has the potential to act as a covalent inhibitor by forming a disulfide bond with the active site Cys134 of the SrtA enzyme. In 5 Ala5 was substituted by *N*-methylglycine (Sar, sarcosine), where the substrate cleavage position at the T–G amide bond is blocked by methylation (Fig. 2a).

The structure of the second series of peptidomimetic inhibitors (6–9) was based directly on the LPETG substrate sequence (Fig. 2a).^32^ The substrate contains a T–G scissile bond which is cleaved by SrtA to form the virulence factor. To increase the stability of this bond against the proteolytic cleavage of the enzyme, the T–G amide bond was modified by *N*-methylation in compound 6 and a G5P mutation in compound 7. Peptide 8 was designed to mimic part of the natural substrate of the enzyme, KSELPETGGEE. Finally, to test the affinity of the enzyme to the substrates' reversed sequence, peptide 9 was introduced.

Based on previous peptidomimetic inhibitors of SrtA,^27–30^ where often a Cbz group was introduced at the N-terminus and our own computational studies (details are discussed later in the manuscript), we sought to more systematically investigate the influence of the introduction of steric bulk at the N-terminus. To this end, a third series of peptidomimetic inhibitors 10–14 was developed, carrying varying degrees of bulk, with an acetyl group in 10 being the smallest and a 2-naphthoyl in 13 the largest. Furthermore, the importance of aromaticity was investigated by including the benzoylated peptide 11 and a peptide bearing a cyclohexyl group in peptide 12.

Finally, the fourth series of inhibitors, peptides 16 and 17 were prepared to investigate the influence of the orientation of the peptide bond between the N-terminal leucine and proline residue in the inhibitor peptides. Peptide bonds can exist in either the *cis*- or *trans*-conformation, where most often the *trans*-conformation is energetically favored, however, the *cis*-conformation can be of importance for biological activity.^33,34^ As earlier shown by us and others, 1,2,3-triazoles can be used as amide-bond surrogates, where the 1,4-disubstituted triazole mimics the *trans*-conformation and the 1,5-disubstituted triazole the *cis*-conformation, furthermore.^33,35^ Additionally, the triazoles are also more stable to proteolytic degradation.^36^ Both the 1,4-disubstituted triazole and 1,5-disubstituted triazole are synthetically easily accessible by CuAAC and RuAAC, respectively.^37,38^

All the peptides (1–17, Fig. 2b) were synthesized by standard Fmoc-based solid-phase peptide synthesis, while additionally peptides 16 and 17 were synthesized using on-resin copper- or ruthenium-catalyzed click chemistry. Peptides were identified by LC-MS followed by HPLC purification and were obtained with at least 95% purity (Table S1 and Fig. S1–S22†). Peptides were further characterized through high-resolution mass spectrometry (Table S1†) with less than 5 ppm deviation from their theoretical calculated mass.

### Sortase A activity and inhibition

In the bacterial cell membrane, SrtA cleaves the Thr–Gly scissile bond of the LPXTG-containing protein and forms a new amide bond with the nucleophilic amino group of the Gly5 moiety of lipid II (Fig. 1).^39^ In the present work, the Abz-LPETGK(Dnp)-NH_2_ substrate was used to monitor the transpeptidation activity of SrtA and its kinetic parameters were determined.^40^ SrtA utilizes the substrate Abz-LPETGK(Dnp)-NH_2_ and H-Gly_3_-OH, producing Abz-LPETGGG-OH and GK(Dnp)-NH_2_ as depicted in Fig. S32a.†

The transpeptidation reaction mediated by SrtA can be monitored conveniently by reverse phase high performance liquid chromatography (RP-HPLC) through analyzing the peak of the released GK(Dnp)-NH_2_ peptide and the consumed substrate peak over time. High-throughput applications of this method for analysis of SrtA inhibition, substrate specificity, and kinetic mechanism are also feasible.^41^ We used a modified protocol from Kruger *et al.* to determine the kinetic parameters of recombinantly expressed SrtA (Fig. S29–S31†).^41^ By incubating SrtA (200 nM) with a varying concentration of its Abz-LPETGK(Dnp)-NH_2_ substrate, Michaelis–Menten kinetics could be applied to obtain the kinetic parameters for the SrtA enzyme (Fig. S32c†). From this study we extracted a *K*_m_ of 2911.3 ± 62.9 μM and a *k*_cat_ of 0.066 ± 0.012 s^−1^, resulting in a *k*_cat_/*K*_m_ of 22.6 M^−1^ s^−1^, which is 16 times higher as initially reported,^41^ confirming that the recombinantly expressed SrtA is sufficiently active and can be used for inhibitory studies.

Before testing our synthetic peptides as potential SrtA inhibitors, their intrinsic stability against cleavage by SrtA was explored, due to their similarity to the SrtA natural substrate (LPXTG motif). The Abz-LPETGK(Dnp)-NH_2_ substrate was used as a positive control and showed that around 26% of the substrate was cleaved by the enzyme after 4 hours (Fig. S33†). This activity of the enzyme was consistent with the calculated kinetic parameters and indicated the feasibility of the test to prove the proteolytic stability for the synthesized peptides. Then, the synthetic SrtA substrate mimics were assayed using the same procedure to confirm their stability against SrtA cleavage. Fortunately, all synthesized peptides appeared to be stable towards SrtA cleavage and could therefore be tested as SrtA inhibitors, the only exception being 4, which showed minor degradation after 4 hours (Fig. S34–S36†).

In order to screen the activity of SrtA inhibitors, a FRET-based functional assay was used which employs an internally quenched fluorescent (IQF) substrate, Abz-LPETGK(Dnp)-NH_2_ (Fig. S37a†).^5^ The synthesized peptidomimetic compounds (1–17) were tested for SrtA inhibition at 200 μM in HEPES buffer (Table 1 and Fig. S37†). 5-((C4-Nitrobenzyl)thio)-1,3,4-thiadiazol-2-amine was used as a reference inhibitor in the experiment.^42^ This compound was reported as a covalent inhibitor and possesses an IC_50_ of around 26 μM, which was in agreement with our results.^42^ Interestingly, 1, 2 and 3, which were designed to mimic the reported PEG_2000_-LPRDA-NH_2_ inhibitor, were not nearly as active as their parent example.^31^ Inhibition for 4 was also rather low, suggesting no covalent inhibition by introduction of the cysteine at this position. The *N*-methylation of Gly in 5 proved to be efficient and manifested high inhibition (69%), making it the strongest inhibitor identified in the first series, while surprisingly, the introduction of Sar in 6 did not result in effective inhibition amongst the second series. 7, on the other hand, was found to be the strongest inhibitor among all four tested series (76%), indicating that replacing the glycine of the scissile T–G bond by either Sar or Pro can be an effective strategy to achieve SrtA inhibition. 8 possessing a high similarity to the natural substrate was not able to efficiently compete with the Abz-LPETGK(Dnp)-NH_2_ substrate with an extent of inhibition of 18%. 9, bearing the reverse sequence of the sorting motif, had moderate inhibitory potency (38%), indicating a loss of recognition when the sequence is reversed.

**Table 1:** Activity data of peptidomimetic SrtA inhibitors; FRET inhibition (200 nM SrtA, 200 μM inhibitor) and IC_50_^a^

Compound	FRET inhibition [%]	IC50 [μM]
Reference (0)	77.1 ± 4.1	—
LPDRA-OH (1)	29.2 ± 2.6	—
LPRDAAdo (2)	30.4 ± 2.8	—
AdoLPRDA (3)	22.6 ± 2.8	—
LPRCA (4)	14.8 ± 2.9	—
LPRDSar (5)	68.5 ± 2.6	18.9 ± 1.2
LPETSar (6)	19.4 ± 2.6	—
LPETP (7)	76.0 ± 8.4	136.3 ± 1.2
KSFLPATGGAE (8)	18.1 ± 2.9	—
GTEPL (9)	37.7 ± 3.1	—
AcLPRDA (10)	20.7 ± 2.9	—
BzLPRDSar (11)	37.2 ± 3.1	—
ChLPRDA (12)	21.6 ± 4.2	—
2NapLPRDA (13)	47.4 ± 1.1 (at 10 μM)	—
CbzLPRDA (14)	17.0 ± 2.8	—
AzARDA (15)	23.9 ± 3.0	—
LtARDA (16)	33.0 ± 3.4	—
LcARDA (17)	23.3 ± 2.9	—
BzLPRDSar (18)	108.4 ± 6.9	57.4 ± 1.1
BzLPETP (19)	15.1 ± 3.8	—
LPRDP (20)	32.3 ± 8.7	—
BLPRDP (21)	31.0 ± 7.6	—
FLPRDA (22)	70.1 ± 5.7	185.3 ± 1.5
BzLPRDF (23)	113.4 ± 26.0	113.8 ± 1.3
LPRDF (24)	42.2 ± 6.0	136.9 ± 1.2
FPRDF (25)	−5.0 ± 5.2	—
FPRDSar (26)	28.3 ± 4.7	—
ELPRDA (27)	26.2 ± 2.0	—
LPETF (28)	−1.8 ± 4.9	—

a The data are reported as mean ± SE%, compounds were measured at 200 μM concentration, with the exception of 2NapLPRDA, 5-((C4-nitrobenzyl)thio)-1,3,4-thiadiazol-2-amine was used as a reference inhibitor. All samples were measured in triplicate. IC_50_ values were corrected based on the peptide's molar extinction coefficient.^43^

Among the N-terminally modified peptides, acetylation did not have a noticeable effect on inhibitory potential, while surprisingly the Cbz-modified peptide was the worst inhibitor in this series. Benzylation of the LPRDA sequence increased the inhibition rate in 11 to 37%, while the non-aromatic counterpart 12 was less active. Due to the incorporated 2-naphthoyl group in 13, which was an inherent fluorescent signal, oversaturation of the detector was reached at 200 μM. Therefore, the single point inhibition was carried out at 10 μM, resulting in a substantial inhibition at 47%. Surprisingly, 14, carrying an N-terminal Cbz-group, which was previously used for covalent peptidomimetic inhibitors of SrtA,^27–30^ showed poor inhibitory potential. Finally, locking the amide bond between Leu1 and Pro2 in either the *cis*- or *trans*-conformation did not result in a significant increase in inhibition, with both 16 and 17 showing poor inhibition (33% and 23%, respectively), furthermore, precursor 15, lacking the locked amide bond, showed a similar potency (24%).

Taken together, these results suggest that the tested peptidomimetic compounds can act as competitive inhibitors at different rates ranging from 17% up to 76% at 200 μM inhibitor concentration. Among the initial panel, two peptides (5 and 7) showed strong inhibition, both of which contain a non-scissile bond in the C-terminal amino acid, in the form of sarcosine in 5 and proline in 7. Furthermore, benzoylation of the N-terminus proved to slightly increase the inhibitory potential as well.

### Second-generation peptidomimetic Sortase A inhibitors

Based on our initial series of peptidomimetic SrtA inhibitors and the understanding gained from our computational analysis (discussed later in the manuscript) a new generation of peptides was designed to more efficiently target SrtA. Firstly, 5 and 7, which showed good initial inhibition, were benzylated to potentially gain a synergistic effect with the increased inhibition observed for the benzylated peptide 11, to obtain peptides 18 (BzLPRDSar) and 19 (BzLPETP). It was observed that the incorporation of sarcosine in the LPRD series resulted in efficient inhibition, while the incorporation of proline in the LPET sequence had the same effect. To complete the range of combinations, peptide 20 (LPRDP) and the benzylated variant 21 (BzLPRDP) were added to the panel as well (Fig. 2c). Furthermore, due to the beneficial influence of N-terminal benzoylation, the LPRDA sequence was extended with a phenylalanine, to probe the general necessity of the benzyl group in peptide 22 (FLPRDA). Additionally, to investigate the effect of benzyl groups elsewhere in the peptide, phenylalanine was introduced at the C-terminus or N-terminus of several sequences (23, 24, 26, 27) or both (25). Finally, to potentially target more polar regions in the SrtA active site by extending further out from the hydrophobic pocket, an N-terminal glutamic acid residue was introduced in peptide 28 (ELPRDA, Fig. 2c). Peptides 18–28 were synthesized by standard Fmoc-SPPS procedures, subsequently purified by RP-HPLC and characterized by LC-MS analysis (Fig. S18–S28†).

Firstly, all second-generation peptides were analyzed regarding their stability towards SrtA, which showed no detectable degradation in any of the samples (Fig. S36†). Henceforth, peptides 18–28 were all tested as SrtA inhibitors (Table 1 and Fig. S37†). In this panel of compounds, benzylation of 5 yielding 18 proved to be an efficient strategy to increase the inhibitory potential of the parent compound, which resulted in full inhibition of SrtA activity at 200 μM concentration. 7, the benzoylated variant of 19, showed poor inhibition (15%), indicating that benzoylation is not a general strategy for these compounds and is heavily sequence-specific. Interestingly, introduction of a proline to the LPRD-sequence or its benzylated variant (20 and 21) did not result in strong inhibition (32% and 31%, respectively), suggesting that the introduction of sarcosine is only beneficial in the LPRD-sequence, while only proline in the LPXT-sequence is tolerated. 22, which contains a phenylalanine residue instead of a benzoyl group to introduce aromaticity on the N-terminus, was shown to be a good inhibitor with 70%, indicating that both modifications can be a viable strategy. Similarly, inhibition increased significantly when 24 (LPRDF) was benzoylated to form 23, which showed full inhibition. However, introduction of phenylalanine proved not to be universally applicable, as 25, 26 and 28 showed poor inhibition or were even completely inactive. Finally, introduction of a negatively charged glutamic acid residue also resulted in poor inhibition of SrtA for 27 (26%) (Table 1 and Fig. S37†).

Subsequently, IC_50_ values were accurately determined for the most potent inhibitors from the initial screen, namely 5, 7, 18, 22 and 23, as well as 24 to directly compare the influence of benzoylation, by varying their concentration between 1 μM and 1500 μM while measuring SrtA inhibition (Table 1 and Fig. S38†). All peptides tested in our panel were shown to have an IC_50_ value below 200 μM. Among these, two peptides (5 and 18) displayed an IC_50_ below 100 μM, with the strongest inhibitor at 18.9 μM for LPRDSar, indicating that this sequence is the most promising for *in vitro* inhibition of the *S. aureus* SrtA enzyme activity. Interestingly, when comparing 23 and 24, benzoylation of the sequence resulted in a slight improvement of inhibitory potential for the benzoylated peptide, while for 5 and 18 the effect was not present. In summary, our series of peptidomimetic SrtA inhibitors appear to be the most potent compounds in this class so far and therefore all six tested compounds were further evaluated for their activity against pathogenic *S. aureus* in growth inhibition studies. Due to the previously mentioned detector saturation, 13 was not evaluated for an IC_50_ measurement. Nevertheless, its significant inhibition at 10 μM suggests an IC_50_ in the range of our strongest inhibitors. Therefore, 13 was evaluated for activity against *S. aureus*.

### Growth inhibition assays

**Fig. 3:**
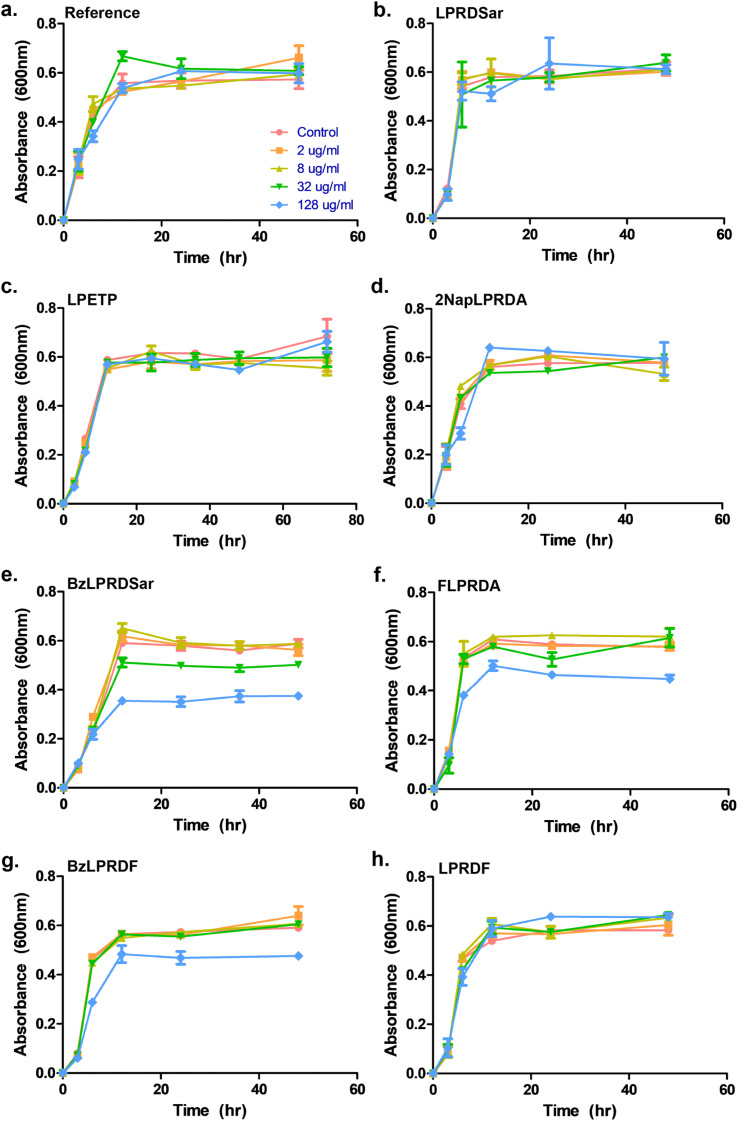
Growth profiling curves of *S. aureus*. The absorbance was measured at 600 nm as a measure of OD for (a) reference; (b) compound 5; (c) compound 7; (d) compound 13; (e) compound 18; (f) compound 22; (g) compound 23; (h) compound 24. All samples were measured in triplicate, error bars are reported as standard error (±SE).

To assess whether our most promising compounds have a possibility of being potential antivirulent compounds, they were tested with pathogenic *S. aureus* bacteria to assess the peptidomimetic inhibitors for their ability to inhibit bacterial growth. Firstly, our compounds were incubated with *S. aureus* bacteria in a 96-well plate in Tryptic Soy Broth (TSB) medium, while we also included the reference compound used in the single-point *in vitro* inhibition studies. So far, the covalent binding thiadiazol reference compound was not tested in this respect, but was one of the most promising compounds identified in an earlier screening campaign.^42^ The inhibitors were added at various concentrations, ranging from 2 μg mL^−1^ to 128 μg mL^−1^, and the absorbance at 600 nm was measured as an indication of their optical density (OD_600_) and by that the bacteria's ability to propagate in liquid media. In the negative control experiments, the bacteria consistently reached an OD_600_ of 0.6 between 6 and 12 hours (Fig. 3). At first glance, it becomes clear that all tested peptidic compounds lacking N-terminal aromaticity are inactive, even at the highest concentration (Fig. 3b, c and h). Furthermore and unfortunately, the thiadiazole inhibitor did not show any activity either (Fig. 3a). Compounds which contained a benzyl ring, either in the form of phenylalanine (22, Fig. 3f) or a benzoylated N-terminus (18 and 23, Fig. 3e and g), did show a clear effect on the plateau of the absorbance at 128 μg mL^−1^. In contrast, 13, with the bulkier 2-naphthoyl group did not have any effect, suggesting it might be too bulky to fit into a SrtA binding pocket, although our computational study (Fig. 5 and Table S3†) predicted an even more significant increase in binding energy for the naphthoyl-compound 13 compared to 18, 22 and 23. Interestingly, the initial growth in the first 6 to 12 hours was not affected by addition of the compounds, rather, beyond the 12 hour timepoint, the absorbance plateau the bacteria reach is lower. This indicates that the bacteria are indeed not being killed, but their growth is being hampered by addition of our peptidomimetic inhibitors. Among the three active compounds, 18 showed the strongest effect, with a 36% decrease in absorbance compared to the control at 48 hours. It was also the only compound which showed an effect at 32 μg mL^−1^. 22 and 23 showed a decrease of 22% and 19% at 128 μg mL^−1^, respectively.

**Fig. 4:**
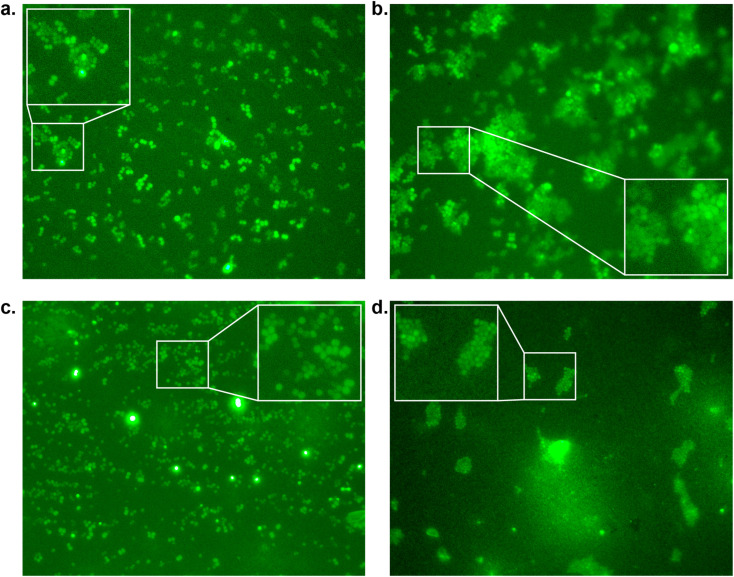
Fluorescence microscopy images of *S. aureus* inhibition by peptidomimetics (stained with Sytox Green at 100× magnification measured at 533 nm) by (a) control; (b) compound 22 at 128 μg mL^−1^; (c) compound 18 at 32 μg mL^−1^, and (d) compound 18 at 128 μg mL^−1^.

To confirm our effect on *S. aureus*, the compounds that showed an effect on the absorbance were also tested using fluorescence microscopy.^44^ To achieve this, *S. aureus* was again incubated with our peptidomimetic inhibitors, specifically, 18, 22, 23 and 24 at 8, 32 and 128 μg mL^−1^ and harvested after 24 hours to ensure the bacteria reach their growth plateau. Peptide 24 was included to confirm the influence of the N-terminal benzyl group. The bacterial cells were permeabilized and their nuclei were labelled using Sytox Green and subsequently their fluorescence was measured quantitatively. A similar trend to the optical density measurements emerged, where 18 had the strongest decrease in fluorescence signal at 128 μg mL^−1^ and a minor effect was seen for 22 and 23 (Fig. S39 and Table S2†). Bacteria incubated with 32 and 128 μg mL^−1^ of 18 as well as 128 μg mL^−1^ of 22 were also imaged using fluorescence microscopy (Fig. 4). In the control sample, planktonic bacteria could be abundantly observed (Fig. 4a). While it was hard to distinguish the microscopy images for the less strong inhibition (Fig. 4b and c), it became evident from the sample with 128 μg mL^−1^ of 18 that visually bacterial growth was affected as well (Fig. 4d) where the bacteria were much more sparsely distributed in the sample. Combined, these results suggest that inclusion of a benzene ring, either in the form of phenylalanine or benzoylation, is required for potential antivirulence effects in *S. aureus*, while also confirming that the peptidomimetic inhibitors do not affect the initial growth, suggesting that they are selectively inhibiting SrtA and not affecting bacterial viability.

Subsequently, our best inhibitor, peptide 18, was tested for its ability to inhibit biofilm formation of *S. aureus*.^45^ Varying concentrations of 18 were incubated with *S. aureus* in Brain Heart Infusion Broth (BHI) medium in a 96-well plate coated with rabbit plasma, to allow the bacteria to form biofilms. After 12 hours of incubation with the peptides, the samples were stained with crystal violet (CV) and then solubilised in ethanol to quantify the biofilms by measuring the absorbance at 595 nm (Fig. S40†). To our delight, at the lowest concentration of 8 μg mL^−1^ tested, a marked decrease of biofilms was detected, with 52% inhibition. Increasing the concentration of the inhibitor to 32 and 128 μg mL^−1^ resulted in 73% and 95% biofilm inhibition respectively, which, within the margin of error, indicates complete biofilm inhibition at the highest concentration tested. These results demonstrate that our peptidomimetic can not only prevent bacterial growth, but it also possesses a potent ability to prevent the formation of pathogenic biofilms. Interestingly, our most potent compounds, 18, 22 and 23, did not show the strongest inhibition in the isolated enzyme assays (Table 1), while inversely, 5 had the lowest IC_50_ in our panel, but did not show any activity in the growth inhibition assays. These results highlight that studies on isolated enzymes can be a decent predictor of *in vitro* activity against bacteria, but show that additional factors are involved in bioactivity, such as the inclusion of a benzene ring in this specific case.

### Computational analysis

To understand the binding of the designed peptidomimetic compounds with the SrtA enzyme as well as to be able to derive meaningful structure–activity-relationships from our experiments, docking studies were performed. Therefore, the recently reported structure of SrtA from *S. pyogenes* (pdb ID 7S51 (ref. 16)) was used to model the SrtA structure from *S. aureus*. Since the SrtA conformation in pdb 7S51 differed somewhat from SrtA *S. aureus* in 2KID,^15^ we decided to create a 7S51-like SrtA *S. aureus* structure and use the ligand position from 7S51 to optimize the SrtA *S. aureus* (SrtA*) substrate binding site for our anticipated docking approach (see the Experimental section for details). In brief, 2KID was aligned with 7S51 and the binding peptide Abz-LPATAG from 7S51 was transferred to 2KID keeping its original binding orientation. Then the ligand was transformed into LPRDSar (ligand 5 in this study) followed by a short refinement molecular dynamic simulation. Thereby, the ligand was fixed in the binding pocket through distance restraints, which mimic the hydrogen bonds between the ligand and the protein in 7S51, allowing the protein to adapt. With respect to our anticipated docking study the highly flexible binding pocket of SrtA is especially problematic, which has been shown by others and also agrees with our own experience from other computational studies.^42,46,47^ The conformational flexibility of the substrate binding pocket was already observable in our short refinement simulation (Fig. S41†), showing that the β6/β7 loop quickly moves from the closed conformation in 2KID towards the open conformation, although a substrate mimicking peptide was present. To account for the highly flexible binding pocket at least to some extent the parent structure as well as the structure of the protein at 92 ns was derived from the refinement simulation, energy minimized and used as the input receptor structure for docking (Fig. S41†).

**Fig. 5:**
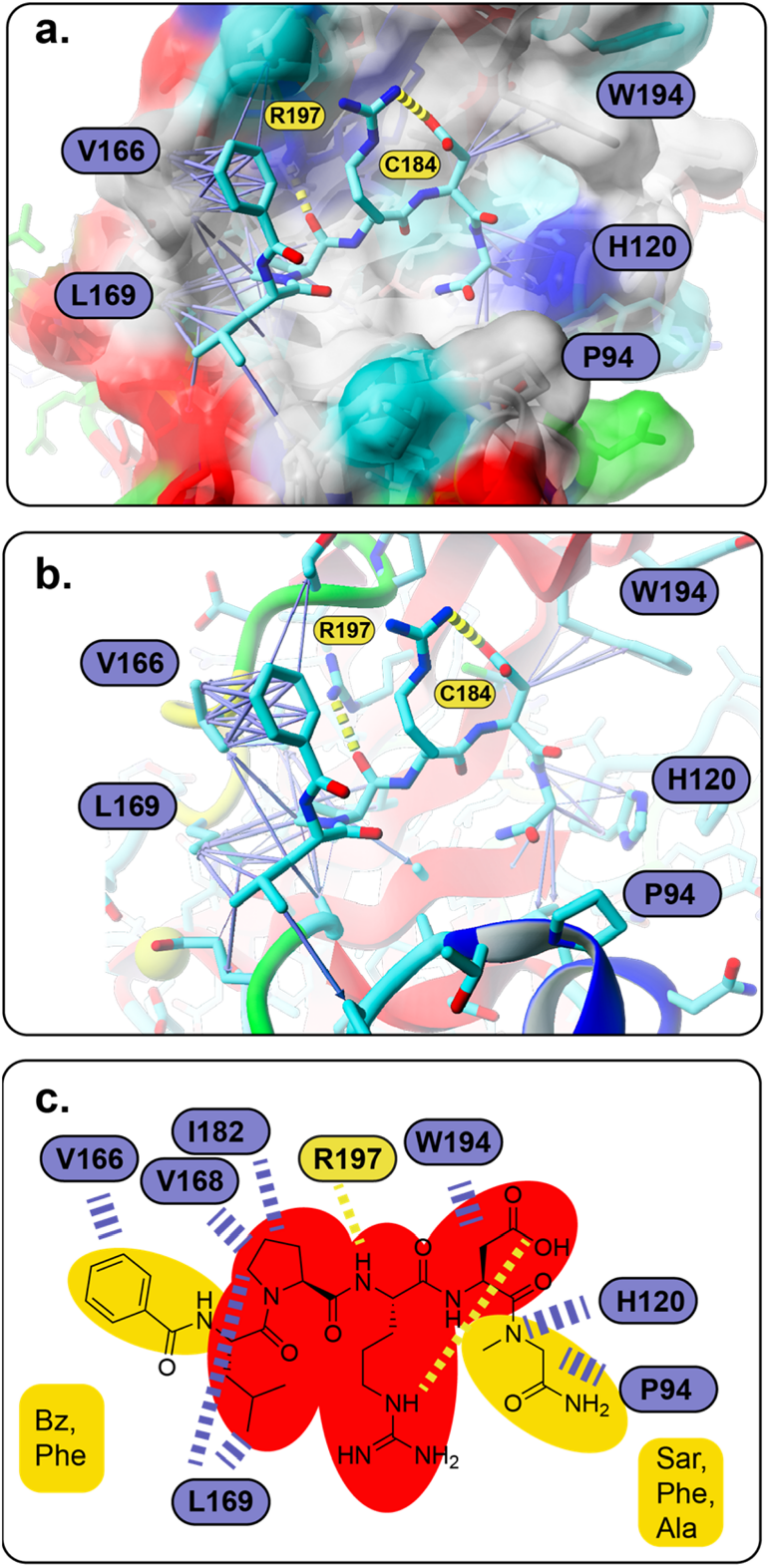
(a and b) Predicted SrtA binding for compound 18, which also resembles the basic binding mode for the majority of compounds in this study (the molecular surface in (a) is colored according to the physicochemical properties of the SrtA residues, red – acidic, dark blue – basic, cyan – polar, green – polar, uncharged, gray – hydrophobic; (a–c) atoms are shown in stick representation and are colored cyan – carbon, blue – nitrogen, red – oxygen, green – sulfur; hydrogen bonds are shown as yellow dashed lines, hydrophobic interactions are presented as purple arrows (dashed lines in c), whose thickness resembles the strength of the interaction) (c) pharmacophore model of LPRD-based SrtA *S. aureus* inhibitors (red – non-variable, yellow – variable positions).

However, all 28 compounds were docked into the two SrtA *S. aureus* structures and in depth analyzed (Table S3†). From the resulting SrtA*-ligand conformations, only those were selected which showed the ligand binding most similar to the substrate as present in pdb 7S51, meaning that the ligand resembles the substrate binding mode. In most but by far not all cases, this conformation was also the best scoring result (Table S3†). In general, the docking score for each ligand in the two SrtA* structures was similar (±0.5 kcal mol^−1^) but also showed large deviations of up to 2.75 kcal mol^−1^ (Table S3,†27). Since, the binding pocket in the SrtA* structure at 92 ns is larger than in the initial structure, especially the larger ligands showed more deviating docking scores. Notably, some ligands were found to show slightly higher scores (≤0.4 kcal mol^−1^) when they are bound in an inverse orientation (Table S3,†7, 18, 22, 23, 24, 26, 28). Although the docking scores did not have any predictable value with respect to our experimental results (Spearman's rank order coefficients are 0.054 and −0.052, Fig. S42†), the analysis of the ligand orientations as well as their hydrophobic interactions (Table S3†) with the receptor allowed us to derive some useful design aspects for an improved ligand binding affinity (Fig. 5). Apart from the large hydrophobic patch, which is typically occupied by the ligands' LP motif in our models, we realized that there are also possibilities for extended hydrophobic interactions around W194 and H120 (Fig. 5a). Thus, these possibilities were successfully explored by creating a more apolar C-terminus, mainly replacing initial Ala with Phe and Sar. In parallel, we successfully improved and increased the strength of hydrophobic interactions around the hydrophobic patch (Fig. 5a), elongating the N-terminus of the ligand by several nonpolar moieties (acetyl, naphthoyl, benzoyl, cyclohexyl and phenylalanine, Table S3†). Hence, these improvements not only resulted in a higher theoretical binding energy (Table S3†), but also yielded potent bioactive compounds, such as compound 18 (Fig. 5a and b). Furthermore, our results allowed us to build a preliminary pharmacophore model (Fig. 5c), which will guide further rational design approaches.

## Conclusions

With the emergence of resistant bacteria, targeting bacterial virulence factors, instead of developing compounds with bactericidal effects, has attracted a lot of attention. If antivirulence treatment is effectively applied, the pathogen becomes unable to form a threat, without killing them, thus leading to a low selective pressure to develop resistance against the antivirulent treatment. Amongst the virulence factors, SrtA is a particularly promising target. SrtA is a membrane-bound transpeptidase enzyme, important for bacterial adhesion to host cells. Previously, inhibition of SrtA in *S. aureus* has been shown to be an effective strategy to prevent formation of pathogenic biofilms.^5^ Furthermore, SrtA inhibition has been directly linked to diseases such as MRSA and septic arthritis, establishing the clinical need for effective treatments relying on SrtA inhibition.^18,20^ Generally, however, inhibitors for SrtA rely on a covalent inhibition mechanism, leading to a high probability of off-target effects towards cysteine containing enzymes. Particularly rare are effective peptidomimetic SrtA inhibitors and here, we aimed to develop novel non-covalent SrtA inhibitors, based on its natural sorting signal (LPETG) and a previously reported sequence (LPRDA).^31^

We have synthesized a panel of 28 potential peptidomimetic SrtA inhibitors, of which several were shown to act as effective competitive inhibitors towards isolated SrtA. Among these, six compounds were found to have an IC_50_ value of below 200 μM, with the compound LPRDSar having the lowest IC_50_ of 18.9 μM. Compounds based on the LPRDA motif seemed to work most effectively, with the introduction of either a non-scissile moiety at the C-terminus, or inclusion of some form of aromaticity in the sequence generally enhancing the inhibitory potential. With these results, our compounds are the strongest *in vitro* peptidomimetic compounds known to date. Furthermore, the six most effective compounds were tested for their potential to inhibit the growth of *S. aureus* bacteria. Here, it was found that compounds containing a phenyl moiety exclusively showed an antivirulent effect, present in the form of a phenylalanine residue or a benzoylated N-terminus. Interestingly, compounds lacking this showed no activity towards the bacteria at all, exemplified by LPRDSar being the strongest *in vitro* inhibitor, while its benzoylated counterpart BzLPRDSar showed the strongest effect against bacterial growth. Furthermore, it was shown that BzLPRDSar was able to prevent biofilm formation at concentrations that make it a potential lead compound. Computational data from our docking studies nicely explain this effect by the strongly increased hydrophobic interactions, when the N-terminus is benzoylated or carries a terminal Phe. Henceforth, experimental and computational data were use to build a preliminary pharmacophore model.

This work is a milestone towards the targeting of an essential virulence factor of Gram-positive pathogenic bacteria, *i.e.*, *S. aureus* SrtA, with possible direct implementation in the prevention of septic arthritis. We believe that with optimization and *in vivo* testing, our peptidomimetic SrtA inhibitors could gain clinical potential, manifesting our strongest inhibitor as a potential lead. The inherent simple structure, non-toxicity of these peptides, combined with their high water solubility and low molecular weight makes them excellent starting points for drug development, in comparison to small organic natural compounds. Additionally, the inclusion of non-scissile bonds, such as sarcosine, in the most promising structure provides protection against host proteases, increasing the probability of the peptidomimetics to reach their targets. Furthermore, the potential of co-administering these SrtA inhibitors with either conventional antibiotics or compounds targeting other virulence factors could increase the clinical application of this strategy further. Taken together, with elucidation of exact structure–activity relationships and *in vivo* testing, antivirulent peptidomimetic SrtA inhibitors could be an excellent starting point to develop treatments counteracting the rapid increase of bacterial resistance to existing drugs.

## Data availability

Data will be made available upon request.

## Author contributions

A. T. and D. T. designed the research and wrote the manuscript. H. A. and J. C. J. H. conducted the research and wrote the manuscript. J. C. J. H., S. R. and I. M. designed and conducted the bacterial experiments. D. T. conducted the *in silico* work.

## Conflicts of interest

There are no conflicts to declare.

## Supplementary Material

SC-014-D3SC01209C-s001
