# Supplementary material for: Substrate-derived Sortase A inhibitors: targeting an essential virulence factor of Gram-positive pathogenic bacteria
Source: Chem Sci. 2023 May 31;14(25):6975–85. doi: 10.1039/d3sc01209c (PMC10306101; doi:10.1039/d3sc01209c)
Supplement: SC-014-D3SC01209C-s001 [file SC-014-D3SC01209C-s001.pdf]

Electronic Supplementary Material (ESI) for Chemical Science.
This journal is © The Royal Society of Chemistry 2023

Supporting Information

Substrate-derived Sortase A Inhibitors: Targeting an Essential Virulence Factor of Gram-positive Pathogenic Bacteria

Helal Abujubara,[a]# Jordi C. J. Hintzen,[a]# Shadi Rahimi,[b] Ivan Mijakovic,[b],[c] Daniel Tietze,[a] and Alesia A. Tietze*[a]

[a] University of Gothenburg, Department of Chemistry and Molecular Biology, Wallenberg Centre for Molecular and Translational Medicine, Kemigården 4, 412 96 Göteborg, Sweden

[b] Chalmers University of Technology, Division of Systems & Synthetic Biology, Department of Biology and Biological Engineering, Kemivägen 10, 412 96, Göteborg, Sweden

[c] The Novo Nordisk Foundation, Center for Biosustainability, Technical University of Denmark, DK-2800, Kongens Lyngby, Denmark

Table of content

Materials and Methods 3
Experimental section 4
Analytical data 9
HPLC and Mass spectrometry 9
Sortase A enzyme expression and purification 19
Biochemical assays 21
Computational Analysis 33
References 35

Materials and Methods

All amino acid derivatives were purchased from Iris Biotech GmbH and used without further purification. ***AmphiSpheres™**, **Novabiochem**®,* and ***TentaGel**®* rink amide resin served as the solid support for the synthesis of carboxyl-terminal peptide amide. ***Novabiochem**®* wang resin served as the solid support for the synthesis of carboxyl group at the C-terminal. All the resins were obtained from Merck. All solvents and reagents were purchased from commercial sources and used as received. For the bioassays: The substrate Abz-LPETGK(Dnp)-NH_2 (0.2784 g, 928 g/mole) was purchased from Bachem AG, Switzerland as a 100 mM stock solution in 3 ml DMSO. H-Gly-Gly-Gly-OH (189.17 g/mol) was purchased from Bachem AG, Switzerland as 50 g powder. The reference compound 5-((4-nitrobenzyl)thio)-1,3,4-thiadiazol-2-amine was prepared as 10 µL of 10 mM stock concentration by Ivana Uzelac at the department of chemistry and molecular biology, Gothenburg University.

Two buffer systems were prepared for the biochemical assays:

1. Tris-buffer (100 ml 300 mM) was prepared using (3.6342 g, 121.14 g/mole) of Tris base, NaCl (0.8766 g, 58.44 g/mole), $CaCl_2$ (0.0555 g, 110.98 g/mole) and Millipore water (100) ml. Ph was adjusted to 7.5 using 1M $NaOH_{(aq)}$ at room temperature 19.5 °C. All components were mixed, and volume filled to 100 ml and filtered using a 90 mm filter paper.
2. HEPES-buffer (100 ml, 50 mM) was prepared using (1.1915 g, 238.3 g/mole) of HEPES (Sigma-Aldrich, Sweden), NaCl (0.8766 g, 58.44 g/mole), $CaCl_2$ (0.0555 g, 110.98 g/mole) and Millipore water (100) ml. Ph was adjusted to 7.5 using 1M $NaOH_{(aq)}$ at room temperature 19.5 °C. Tween 20 (50 µL) was added as 0.05% v/v to prevent foam formation. All components were mixed, and volume filled to 100 ml using a 90 mm filter paper.

Analytical TLC was performed using silica gel plates with C18 functionality and visualized under UV light at (254 nm).

The *E. Coli SrtA* gene used for expression codes for a protein product of 156 amino acids with an N-terminal His6-tag. The Ampicillin-resistant host strain *E. coli* BL21 transfected with pET21b plasmid was prepared lab at the chemistry and molecular biology department of Gothenburg University (Medicinaregatan 5c, 41390 Göteborg). Luria broth (LB) medium (Tryptone 10 g/L, Yeast extract 6 g/L, NaCl 5 g/L, and MQ-H_2O) was prepared to perform the expression of SrtA. Tryptone, Yeast extract, and NaCl were purchased from *Sigma-Aldrich®.* DiluPhotometer was used to measure the (OD_{600nm}) which is the optical density at the wavelength of 600 nm.

SDS-PAGE analysis: Mini-protein® Tris-Tricine precast Gels were used for electrophoretic analysis with running buffer containing (100mM tris, 100mM tricine plus 0.1% SDS). All samples were incubated at 90 °C for 5 min in the presence of 4 µl of 4X LDS sample buffer. The gel was done at 100 v for *ca.* 100 min. The gel was stained with Coomassie blue stain and de-stained in 10% acetic acid aq.solution before scanning. Precision plus protein dual Xtra standards™ was used as a protein ladder. For the reducing gel, B-Mercaptoethanol was added to the sample buffer as 5% v/v.

Fast protein liquid chromatography (FPLC): HiLoad_16/60_Supperdex_75_prep_grade size exclusion column was used as a stationary phase for the protein purification. The column was equilibrated with tris buffer and isocratic elution of this buffer was used with 1.0 ml/min flow rate and 7.9 ph. The column temperature was set to 4 °C and max pressure to 0.5 MPa. UV-detection was measured at 280 nm wavelength. The sample pump was used for loading and the machine was equipped with automatic round fraction collector F9-R.

NanoDrop® ND-1000 UV-Vis spectrophotometer was used to determine the concentration of the protein samples at 280 nm wavelength with molar absorptivity (ε) equals to 14440 and 17.7 KDa expected molecular weight.

Experimental section

Organic synthesis

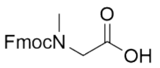

***N*-Fmoc-*N*-methylglycine (Fmoc-Sarcosine)** Synthesis was performed according to previously published methods.[1] Briefly, Sarcosine (1.425 g, 16 mmol) and Na_2CO_3 (1.695 g, 16 mmol) were dissolved in acetone/H_2O (15 mL, 1:1.5). Fmoc-OSu (5.397 g, 16 mmol) was added over a period of 1 h while the pH was kept at 9-10 by the addition of 1M aq. Na_2CO_3. After stirring overnight, EtOAc (40 mL) was added and the mixture was acidified with 6N HCl. The organic layer was separated and washed with H_2O (4x, a total of 80 mL), dried with $MgSO_4$, and concentrated to 3 mL. Crystallization with $CHCl_3$ yielded 2g (6.4 mmol, 40% yield) as pure white crystals. The purity was assessed by analytical HPLC (>95%). MS(LC/MS-ESI), m/z (found): 312.35 $[M+H]^+$, m/z (calc.): 312.15 $[M+H]^+$.

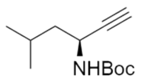

(3*S*)-3-(*N*-Boc)-amino-5-methyl-1-hexyne (Boc-Leu-alkyne). Boc-Leu-aldehyde (500mg, 2.3 mmol) was dissolved in MeOH (15 mL). Subsequently, K_2CO_3 (550 mg, 3.9 mmol) and the Bestmann-Ohira reagent (410 mg, 2.1 mmol) were added to the resulting solution and stirred for 24h at room temperature.[2] The solvents were evaporated under vacuum, and the resulting crude mixture was dissolved in EtOAc/MQ-H_2O (40 mL, 1:1). Thereafter, the layers were separated, and the organic layer was washed with MQ-H_2O and dried with $MgSO_4$, filtered, and removed under vacuum, which resulted in a yellow oil, and was used without further purification.

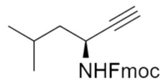

(3*S*)-3-(*N*-Fmoc)-amino-5-methyl-1-hexyne) (Fmoc-Leu-Alkyne). The Boc-Leu-alkyne crude was dissolved in TFA/CH_2Cl_2 (5 ml, 1:1) and stirred for 90 min at room temperature. The solvents were evaporated resulting in a yellow oil. The oil was dissolved in Acetone/MQ-H_2O (10 ml, 1:1) and Na_2CO_3 (305 mg, 2.85 mmol) was added. The reaction mixture was alkalized to pH 9-10 with 1M aqueous Na_2CO_3 and Fmoc-OSu (900 mg, 2.15 mmol) was slowly added under vigorous stirring. The resulting solution was stirred at room temperature for 24h. Then, EtOAc (20ml) was added and 6M HCl was added to acidify the solution. The organic layer was separated, washed with MQ-H_2O (4x10ml) and dried with $MgSO_4$. The organic solvent was filtered off, and removed under vacuum, which resulted in a brown to orange oil. The oil was purified with flash column chromatography using gradient elution (pentane/EtOAc, 0 to 30%). The pure Fmoc-Leu-alkyne eluted at around 28% EtOAc (163 mg, 0.48 mmol, 21% yield over three steps). ^{1}H NMR (400 MHz, CD_2Cl_2): d=7.79 (d, *J*=6.8 Hz, 2H), 7.61 (d, *J* = 7.5 Hz, 2H), 7.37 (m, 4H), 4.96 (s, 1H), 4,5 (m, 3H), 4.23 (t, *J* = 6.9 Hz, 1H), 2.34 (d, *J* = 2.3 Hz, 1H), 1.77 (m, 1H), 1.55 (t, *J* = 7.4 Hz, 2H), 0.94ppm (d, *J* = 6.6 Hz, 7H); ^{13}C NMR (101 MHz, CD_2Cl_2) d=155.2, 143.9, 141.3, 127.6, 127.0, 125.0, 119.9, 83.5, 70.7, 66.6, 53.9, 47.2, 44.8, 41.6, 24.9, 22.2, 21.8 ppm; MS(LC/MS-ESI), m/z (found): 356.1570 $[M+Na]^+$, m/z (calc.): 356.1627 $[M+Na]^+$.

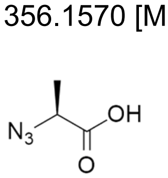

(2*S*)-2-Azido-propanoic acid: A solution of sodium azide (250 mg, 3.1 mmol) was dissolved in distilled MQ-H_2O (13 mL) with CH_2Cl_2 (22 mL) and cooled on an ice bath. Triflic anhydride (7.7 mL, 1M solution in CH_2Cl_2) was added slowly over 5 min with stirring continued for 2 h. The organic layer was separated and the aqueous layer was extracted with CH_2Cl_2 (2 × 20 mL). The organic fractions were combined and washed once with saturated Na_2CO_3 solution and used without further purification. A mixture of L-alanine (35.7 mg, 0.4 mmol), K_2CO_3 (81.8 mg, 0.6 mmol), $CuSO_4$ pentahydrate (1.4 mg, 5.6 µmmol) was dissolved in 25 mL H_2O/methanol solution (1:2, v:v), and the prepared triflyl azide in CH_2Cl_2 was added. The mixture was stirred at room temperature overnight. Subsequently, the organic solvents were removed under reduced pressure and the aqueous

slurry was diluted with H_2O (50 mL), acidified to pH 6 with conc. HCl, diluted with 50 mL phosphate buffer (0.25 M, pH 6.2), and extracted with EtOAc (4 × 20 mL) to remove the sulfonamide by-product. The aqueous phase was then acidified to pH 2 with conc. HCl. The product was obtained from another round of EtOAc extractions (3 × 80 mL). These EtOAc extracts were combined, dried over $MgSO_4$, and evaporated to dryness giving a pure yellow oil with no need for further purification 40 mg (0.34 mmol, 11% yield over two steps). ^{1}H NMR (400 MHz, $CDCl_3$) δ 6.13 (s, 1H), 4.02 (q, *J* = 7.2 Hz, 1H), 1.54 (d, *J* = 7.2, 0.6 Hz, 3H); ^{13}C NMR (101 MHz, $CDCl_3$) δ 77.21, 57.00, 16.68.

Synthesis and purification of peptides

Peptides were chain assembled using Rink amide resin either automated or manually. For automated synthesis, an Intavis MultiPep CF was used. Amino acid couplings were carried out with the molar ratio of (4):(4):(8) of (Fmoc-protected amino acid):(HBTU):(NMM) at room temperature for 60 minutes and deprotection was achieved in 20% (v/v) piperidine in DMF for 15 minutes at room temperature. After coupling, peptides were capped with 5% acetic anhydride in DMF (v/v) for 5 minutes. Manual amino acid couplings were carried out with the molar ratio of (3):(3):(4.5) of (Fmoc-protected amino acid):(HATU):(DIPEA) at room temperature for 60 minutes and deprotection was achieved in 20% (v/v) piperidine in DMF for 30 minutes at room temperature. N-terminal acetylation was achieved by reacting with acetic anhydride (10 eq.) and TEA (10 eq.), benzoylation with benzoyl chloride (10 eq.) and TEA (10 eq.), cyclohexanoylation with cyclohexanecarbonyl chloride (10 eq.) and TEA (10 eq.), naphtoylation with 2-naphtoyl chloride (10 eq.) and TEA (10 eq.) in DCM for 1 hour. Incorporation of the Cbz group was achieved by reacting with benzyl chloroformate (10 eq.) and DIPEA (10 eq.) for 3 hours at 0 °C. To generate the triazoles on resin, the synthetically prepared (2*S*)-2-azido-propanoic acid (3 eq.) was coupled twice with HATU (2.9 eq.) and DIPEA (4.5 eq.) for 1 hour. Subsequently, the resin was split into two portions and the *trans*-substituted triazole was synthesized by reacting with prepared Fmoc-Leu-Alkyne (4 eq.), $CuSO_4{\cdot}5H_20$ (20mol%), sodium ascorbate (32 eq.) and DIPEA (6 eq.) in DMF for 5 hours at 60 °C under microwave irradiation using a Biotage Initiator+ microwave synthesizer (Biotage Sweden AB, Uppsala, Sweden), followed by washing with 0.5% sodium diethyldithiocarbamate in DMF, DCM and DMF, and subsequent Fmoc deprotection. The *cis*-substituted triazole was synthesized by reacting with prepared Fmoc-Leu-Alkyne (4eq.) and Cp*RuCl(cod) (20mol%) in DMF for 5 hours at 60 °C under microwave irradiation, followed by washing with 0.5% sodium diethyldithiocarbamate in DMF, DCm and DMF, and subsequent Fmoc deprotection. Peptides proceeded to standard cleavage from resin using a mixture of TFA (95%), H_2O (2.5%) and TIPS (2,5%) for 3 hours at room temperature, while for cysteine-containing peptides EDT (2.5%) was added to the cleavage cocktail additionally. TFA was removed using N_2 and the resultant residue suspended in ice-cold diethyl ether. The mixture was then centrifuged (5 min, 4600 RPM) after which the supernatant was decanted into the waste. The remaining solid was washed twice by ice-cold diethyl ether and subjected to purification. For the purification and characterization of peptides, two eluent systems were used. Mobile phase A was 0.1% TFA in MQ-H_2O, mobile phase B was 0.1% TFA in ACN and detection was done at 214 nm. The crude peptide was dissolved in a mixture of ACN in H_2O and purified by semi-preparative HPLC using a Waters 600 system (Waters, Milford, MA, USA) equipped with a C18 column (MultoKrom 100 – 5 C18, 5 µm particle size, 100 Å pore size, 250 x 20 mm, CS Chromatographie Service, Langerwehe, Germany) and a gradient of mobile phase A and mobile phase B from 5-10% B to 20-50% B over 45 min at 8 mL/min, depending on the specific peptide. Analytical RP-HPLC was carried out on a Waters XC e2695 system (Waters, Milford, MA, USA) employing a Waters PDA 2998 diode array detector equipped with a ISAspher 100-3 C18 (C18, 3.0 µm particle size, 100 Å pore size, 50×4.6 mm, Isera GmbH, Düren, Germany) at a flow rate of 2 mL/min using various gradients. The molecular weight of the purified peptides was confirmed by ESI mass on a Waters Synapt G2-Si ESI mass spectrometer equipped with a Waters Acquity UPLC system using a Xela C18 column (C18, 1.7 µm particle size, 80 Å pore size, 50×3.0 mm, Isera GmbH, Düren, Germany).

Protein expression and purification

The *E. coli* strain transfected with the recombinant plasmids was grown overnight in LB medium containing 100 µg/mL Ampicillin at 37 °C with constant shaking at 177 rpm. The bacterial culture was propagated into 1L LB at 37 °C until the OD_{600} reached 0.46 and then incubated with 0.5 mM IPTG (isopropyl β-d-thiogalactoside) at 37 °C for 3 h to induce sortase expression. After the completion of incubation, the cells were harvested by centrifugation at 8000 × g for 15 min and stored at −80 °C until use. For protein purification, the cells were suspended in 35 mL lysis buffer (50 mM Tris·HCl, pH 8 at 21 °C, 150 mM NaCl, and 25 mM imidazole), lysed by high-pressure homogenization and then centrifuged at 25000 × g for 30 min at 10 °C. A LM20 Microfluidizer was used as a homogenizer for cell lysis with 25 kPsi air pressure at 4 °C**.** The lysate supernatant was then loaded into a gravity-flow column filled with 2 ml Ni Sepharose 6 Fast Flow resin and incubated for 30 min at 400 rpm and 4 °C. Then, the Sepharose was washed with four column volumes of lysis buffer to remove intracellular contaminating proteins. The Sortase A protein containing N-terminal His-tag was eluted with five column volumes of elution buffer (50 mM Tris·HCl, pH 8 at 21 °C, 150 mM NaCl, and 500 mM imidazole). The protein was concentrated via centrifugal filtration using Vivaspin with a molecular weight cutoff of 10 kDa. The supernatant fluid, flow through and the SrtA protein were analyzed by SDS-PAGE. Following gel analysis, the protein was desalted using a PD-10 column containing 8.3 ml of Sephadex G-25 medium. The column was equilibrated with 25 ml of Tris buffer (30 mM Tris, 150 mM NaCl, pH 8 at 21 °C). For optimization experiments, the protein was purified by Fast protein liquid chromatography (FPLC) through loading into a HiLoad 16/60 Superdex 75 column. The fractions collected from (FPLC) chromatography were checked by using a NanoDrop spectrophotometer and analyzed in SDS-PAGE (**Figures S29-S31**). Both reducing and non-reducing gel electrophoresis analyses were made for the eluted fractions. The pure SrtA protein was checked by LC-MS. Mass spectrum was recorded in positive ion mode over an m/z range of 50–2000. ESI+ for $C_{787}H_{1238}N_{220}O_{241}S_4$**;** calculated 17766.06 Dalton, found 1790.68 m/z as $[M+10H]^{10+}$, 1628.09 m/z as $[M+11H]^{11+}$ and 1492.59 m/z as $[M+12H]^{12+}$. The protein was divided into a set of aliquots as stock solutions in Tris buffer (30 mM Tris and 150 mM NaCl). The aliquots of the enzyme were stored at -80°C until further use.

Kinetic analysis of the Sortase A activity

The kinetic assay protocol was performed based on the assay published by Kruger *et al.*[3] The biochemical assay was carried out in 210 µL reaction mixtures at pH 7.5 containing Tris buffer (300 mM Tris, 150 mM NaCl and 5 mM $CaCl_2$), Gly_3 (2 mM), SrtA (200 nM), and varying concentrations of Abz-LPETGK(Dnp)-NH_2 (from 30 to 900 µM). The reaction mixture was incubated for 6 hrs at 37 °C. Injections of 20 µl were taken each hour automatically and analyzed using RP-HPLC. The peptides were separated using a 30 to 50% linear gradient of eluent B over 5 min. The substrate peaks were detected by absorbance at 355 nm, and the extent of transpeptidation was calculated from the decrease of the area of the substrate peak over time. The peak area was converted to concentration units using a calibration curve of Abz-LPETGK(Dnp)-NH_2 as the standard (**Figure S32b**). Duplicate measurements were taken for each data point. Microsoft Origin 2019 was used to calculate the kinetic constants *Km*, *V*max, and *k*cat from the curve fit for the Michaelis-Menten equation. The data were reported as mean ± SE%.

Chromatographic assay of the peptide's stability

The cleavage assay set-up and protocol were modified from a previously published assay.[3] The assay was performed in 200 µl reaction mixtures. The cleavage of the synthetic peptides was done by incubating 90 µL of SrtA (270 nM final concentration) with 90 µL of compound solution (270 µM) in Tris buffer (300 mM Tris, 150 mM NaCl, 5 mM $CaCl_2$, pH 7.5) for 4 h at RT. Gly_3 solution in Tris buffer (20 µL, 2 mM) was added to the mixture to accomplish the cleavage. Peptide substrate solution in Tris buffer (33.75 µM Abz-LPETGK(Dnp)-NH_2) was used in the experiment as a positive control and tested for cleavage in the same protocol. Samples of 50 µl were taken from the reaction mixture each hour and quenched by the addition

of 25 µl 1M HCl to stop the enzymatic activity. The samples were analyzed by RP-HPLC and separated using a linear gradient from 0% to 40% of eluent B over 10 min. Dnp-containing peaks in the substrate were detected by measuring the absorbance at 355 nm while the synthetic peptides peaks were detected at 214 nm. The extent of cleavage was monitored over 4 hrs by measuring the product/substrate peaks ratio through integrating the areas under the HPLC trace. If cleavage occurred, the detected peaks were checked by ESI-MS. GK(Dnp)-NH_2 and Abz-LPETGK(Dnp)-NH_2 were detected by absorbance at 355 nm with retention times of 7.19 minutes and 9.97 minutes, respectively.

Fluorescence resonance energy transfer assay

For the FRET assay, the compound 5-((4-nitrobenzyl) thio)-1,3,4-thiadiazol-2-amine was used as a reference and prepared by dissolving 3 µL of the available stock (10 mM) in 96 µL HEPES and 1% DMSO to afford a 300 µM stock concentration for the assay. This reference compound has a reported IC_{50} value of 26 ± 0.7 µM.[4]

The fluorometric assay protocol has been previously used for screening of SrtA inhibitors.[5] The FRET assay was performed with an internally quenched fluorogenic (IQF) substrate to determine the inhibitory activity of the synthesized peptidomimetics. The assay was performed in a 30 µl reaction mixture in HEPES buffer (50 mM HEPES, 150 mM NaCl, 5 mM $CaCl_2$, 0.05% Tween20, pH 7.5). SrtA solution (200 nM) was preincubated with compounds (200 µM) for 15 min at 22 °C followed by the addition of the substrate solution (25 µM Abz-LPETGK(Dnp)-NH_2, 5 mM H-Gly_3-OH, final assay concentration) in the same buffer. The cleavage of the Abz/Dnp-substrate was quantified by an increase in the fluorescence signal. The fluorescence signal was monitored each minute for 45 minutes and plotted to obtain the fluorescence rate as a line curve. The assay was performed in 384 well Corning NBS microplates (flat bottom, no lid, low flange, non-binding surface, non-sterile, white polystyrene). Fluorescence intensity was measured at emission and excitation wavelengths of 320 and 420 nm, respectively using a Spectramax iD5 plate reader (Molecular Devices, San Jose, CA, USA). Triplicate measurements were taken for each data point to ensure the reproducibility of the results. The average value of the fluorescence signal readouts was plotted against time over 60 min for each compound to produce a line curve using Origin. The reference compound was used in the experiment to act as a positive control. The slope of each compound curve (S_x) and the slope of the substrate (S_0) were extracted using the linear fit function in Microsoft Origin 2019. The percent inhibition was calculated according to the following equation: inhibitory rate = $(1 – S_x/S_0) \times 100\%$. For IC_{50} values, S_x values at different concentrations were plotted against log transformed concentration values and fitted with a sigmodial fit using the DoseResp function using Origin. The bottom asymptote of the dose-response curve was fixed at 0. The data were reported as mean ± SE%.

Growth Inhibition Assays

For the growth profiling experiments the *S. aureus* strain CCUG10778 was cultured overnight in sterile TSB medium and diluted 1:100 by adding 1 µL into TSB medium in a sterilized, clear polystyrene 96-well plate to a final assay volume of 100 µL. Subsequently, peptides were added to the bacteria from filter sterilized stocks in MQ-H_2O to give 2, 8, 32 and 128 µg/ml final concentrations. Then, bacteria were incubated at 37 °C for 3, 6, 12, 24, 36 or 48 hours and their absorbance was measured at 600 nm using a Varioskan Lux microplate reader (Thermo Fisher Scientific, Waltham, MA, USA). All individual samples were carried out in triplicate.

For the fluorescence experiments *S. aureus* was cultured overnight in sterile TSB medium and diluted 1:250 by adding 2.5 µL bacterial culture into TSB medium in sterilized 1.5 mL Eppendorf tubes to a final volume of 500 µL. Subsequently, peptides were added to the bacteria from filter sterilized stocks in MQ-H_2O to give 8, 32 and 128 µg/ml final concentrations. Then, bacteria were incubated at 37 °C for 24 hours and centrifuged for 3 minutes at 3000 rpm in an Eppendorf Microstar 30r centrifuge. The supernatant was discarded and the cells were washed once with sterilized PBS and centrifuged. 100 µL PBS-T (PBS + 0.1% Triton-X) was added to the cells for 15 minutes to induce cell membrane permeabilization. After removal of the PBS-T by centrifugation, 100 µL of 5 µM Sytox Green (Invitrogen, Waltham, MA, USA) solution in PBS

was added and the cells were stained for 30 minutes, centrifuged and washed with PBS twice. For the fluorescence measurements, the cells were resuspended with 100 µL of PBS and added in a sterilized, white plastic polystyrene 96-well plate and their fluorescence was measured by exciting the samples at 488nm and measuring the fluorescence intensity at 533 nm in a microplate reader. All individual samples were carried out in duplicate. For the fluorescence microscopy experiments, the cells were treated in the same, but resuspended in 10 µL of PBS and 5 µL of bacterial suspension was spotted on a glass target plate dipped in a melted solution of 1.2% agarose in Tris-HCl (50 mM) to ensure fixation of the sample. The sledes were covered by a cover glass and observed on an Axio Imager Z2m fluorescence microscope (Zeiss, Jena, Germany) at 100 times magnification at the emission wavelength of 533 nm.

Biofilm inhibition assays

For the biofilm inhibition experiments the *S. aureus* strain CCUG10778 was cultured overnight in sterile BHI broth. A sterilized, clear polystyrene 96-well plate was prepared by coating with plasma by addition of 20% of final assay volume rabbit plasma to the wells and incubating overnight at 4 °C. Then, the cultured strain was diluted 1:100 by adding 2 µL into BHI medium on the plasma coated plate to a total volume of 200 µL. Subsequently, peptides were added to the bacteria from filter sterilized stocks in MQ-H_2O to give 8, 32 and 128 µg/ml final concentrations and incubated for 12 hours at 37 °C without shaking. The supernatant was removed and the wells were rinsed with PBS and 100 µL of 1% crystal violet solution (diluted in PBS) was added and the biofilms were stained for 10 minutes. The wells were washed with PBS twice and 200 µL of a 95% ethanol solution in water was added to solubilize the cells for 30 minutes. 100 µL was transferred into a new 96-well plate and the absorbance measured at 595 nm using a Varioskan Lux microplate reader. All individual samples were carried out in triplicate.

Computational Studies

All computational studies and analysis steps were performed employing the YASARA molecular modeling program.[6, 7]
Molecular graphics were created with YASARA (www.yasara.org) and POVRay (www.povray.org).

Docking
Docking was performed using VINA[8] (default parameters) and the best scoring and/or fitting results from 25 independent docking runs were further analysed. Docking was restricted to the substrate binding pocket of SrtA. Ligands and receptor residues were kept flexible during the docking runs.

Receptor structure generation
The structure of SrtA S. aureus was derived from pdb 2kid and was structurally aligned with pdb 7S51.[9, 10] Then the binding peptide Abz-LPATAG from 7S51 was transferred to 2kid keeping its original binding orientation and then transformed into LPRDSar. The resulting protein structure was geometry optimized with the YASARA YAPAC module using semi-empirical, quantum chemical methods followed by an energy minimization employing the Yasara2 force field[11] and refined in a 110 ns molecular dynamic simulation. Thereby, the ligand was fixed in the binding poket through harmonic restraints (O Leu 2, NH1 Arg 197; O Pro 3, NE Arg 197; O Asp 5, N Cys 184; O Asp 5, N Thr 121) which mimic the hydrogen bonds between the ligand and the protein in 7S51. The parent structure as well as the structure of the protein at 92 ns were derived from the refinement simulation, energy minimized and used as the input receptor structure for docking.
Energy minimizations and *molecular dynamic simulations* of the inhibitor-SrtA complex were performed as all-atom molecular dynamics simulation in explicit water (TIP3P) using the PME method[12] to describe long-range electrostatics at a cut-off distance of 8 Å at physiological conditions (0.9% NaCl, pH 7.4[13]) at constant temperature (298 K) using a Berendsen

thermostat and constant pressure (1 bar). Charged amino acids were assigned according to the predicted pKa of the amino acid side chains by Ewald summation and were neutralized by adding counter ions (NaCl).[13] In order to increase the simulation performance a multiple time step algorithm together with a simulation time step interval of 2.5 fs.[7] Simulation snapshots were saved every 100 ps. Time traces were computed from these snapshots.
Energy minimizations were performed by simulated annealing including optimization of the hydrogen bond network[14] and equilibration of the water shell until system convergence was achieved (<0.05 kJ/mol/200/steps).

Analytical data

HPLC and Mass spectrometry

Table S1. Analytical data for the peptides

Number	Peptide	Calculated Mw [g/mol]	Experimental Mw[(d)] [g/mol]	ppm	tR [min]
**1**	H_2N-LPRDA-OH	571.3204	571.3232	4.9	6.53
**2**	LPRDA-Ado[(a)]	715.4103	715.4074	-4.1	7.12
**3**	Ado-LPRDA	358.2090[(e)]	358.2111[(e)]	5.9	8.92
**4**	LPRCA	558.3186	558.3164	-4.1	4.12
**5**	LPRD-Sar[(b)]	570.3364	570.3342	-3.9	4.69
**6**	LPET-Sar	529.2986	529.3016	5.3	3.57
**7**	LPETP	555.3142	555.3171	5.2	2.35
**8**	KSFLPATGGAE	538.7910[(e)]	538.7929[(e)]	3.3	5.32
**9**	GTEPL	515.2830	515.2833	0.6	3.48
**10**	AcLPRDA	612.3469	612.3475	1.1	3.04
**11**	BzLPRDA	674.3626	674.3625	-0.2	5.27
**12**	ChLPRDA	680.4095	680.4097	0.4	5.62
**13**	2NapLPRDA	724.3782	724.3823	5.7	6.89
**14**	CbzLPRDA	374.1880[(e,f)]	374.1876[(e,f)]	-1.1	4.06
**15**	AzARDA[(c)]	457.2271	457.2271	0	1.64
**16**	LtARDA	568.3320	568.3337	3.0	2.14
**17**	LcARDA	568.3320	568.3342	3.8	2.16
**18**	BzLPRDSar	674.3626	674.3655	4.3	5.22
**19**	BzLPETP	659.3405	659.3437	4.9	5.13
**20**	LPRDP	596.3520	596.3528	1.3	1.87

**21**	BzLPRDP	700.3782	700.3819	5.3	5.10
**22**	FLPRDA	717.4048	717.4089	5.7	1.40
**23**	BzLPRDF	750.3939	750.3900	-5.2	4.78
**24**	LPRDF	646.3677	646.3707	4.6	3.06
**25**	FPRDF	680.3520	680.3545	3.6	3.40
**26**	FPRDSar	604.3207	604.3233	4.5	1.84
**27**	LPETF	605.3299	605.3304	0.8	3.19
**28**	ELPRDA	699.3818	699.3790	4.0	1.64

(a) Ado: Amino -3,6-dioxaoctanoic acid.
(b) Sar: 2-(methylamino) acetic acid.
(c) AzA: Azidoalanine
(d) Mass peaks detected as $[M+H]^+$
(e) Double charged peak
(f) Mass peak detected as $[M+K]^+$

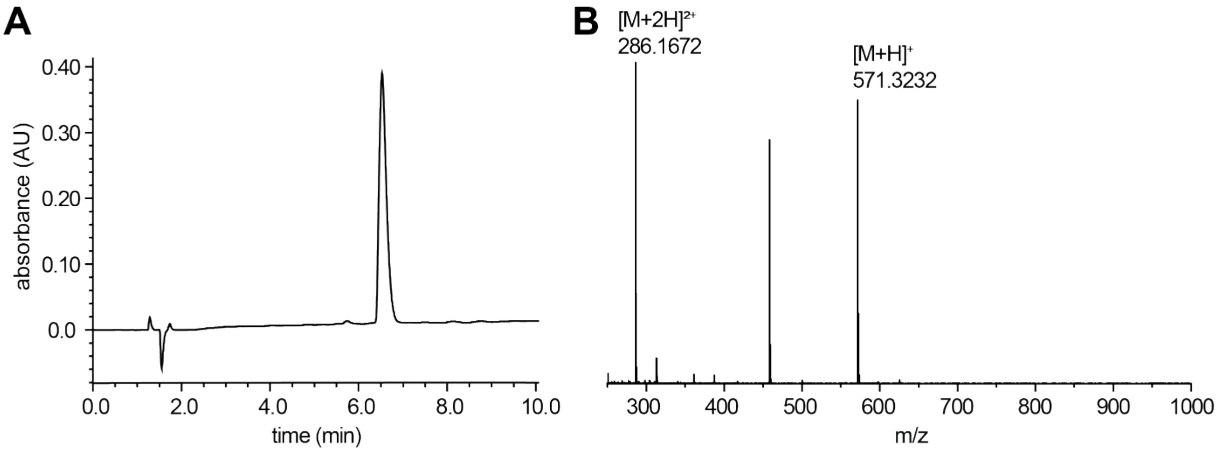

Figure S1. A) HPLC chromatogram (gradient from 5 to 25% ACN in water over 15 min at 1 ml/min, detection at 214 nm) and B) high resolution mass spectrum for peptide 1 H_2N-LPRDA-OH.

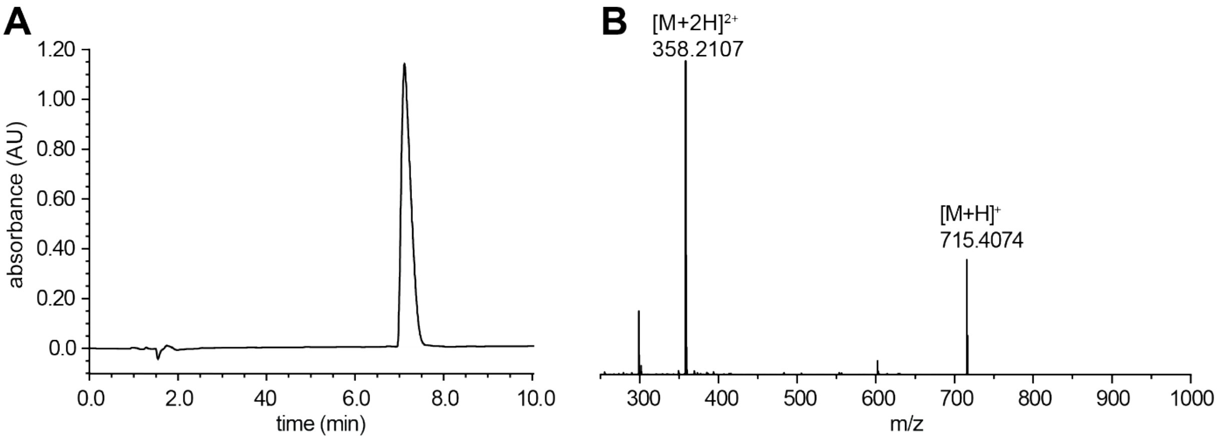

Figure S2. A) HPLC chromatogram (gradient from 5 to 25% ACN in water over 15 min at 1 ml/min, detection at 214 nm) and B) high resolution mass spectrum for peptide 2 LPRDA-Ado.

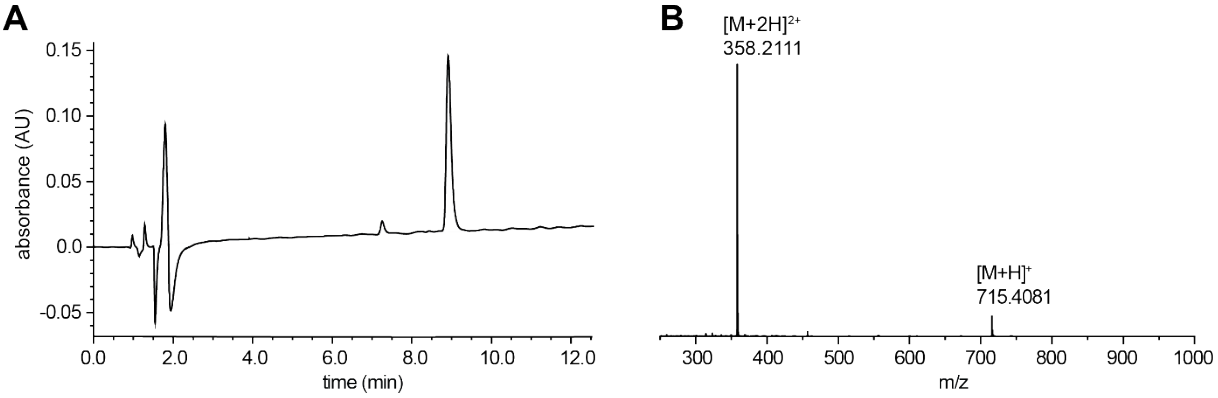

Figure S3. A) HPLC chromatogram (gradient from 5 to 25% ACN in water over 15 min at 1 ml/min, detection at 214 nm) and B) high resolution mass spectrum for peptide 3 Ado-LPRDA.

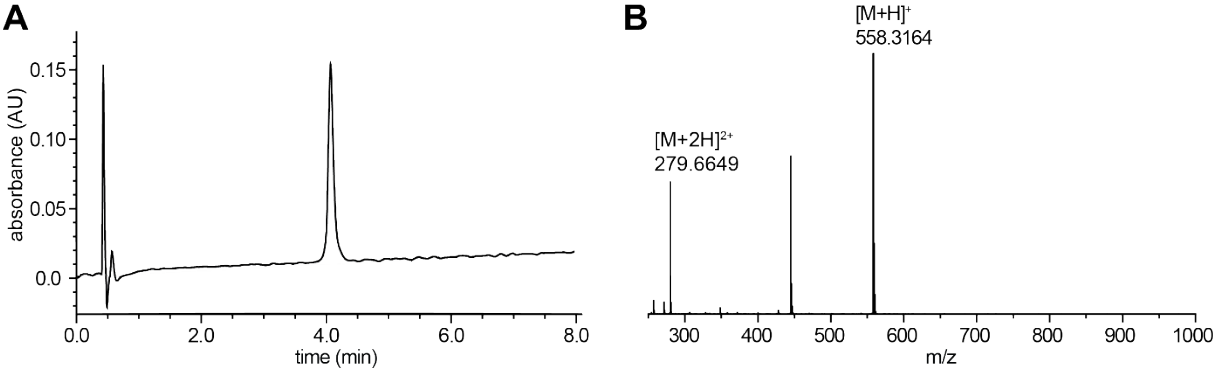

Figure S4. A) HPLC chromatogram (gradient from 5 to 25% ACN in water over 10 min at 2 ml/min, detection at 214 nm) and B) high resolution mass spectrum for peptide 4 LPRCA.

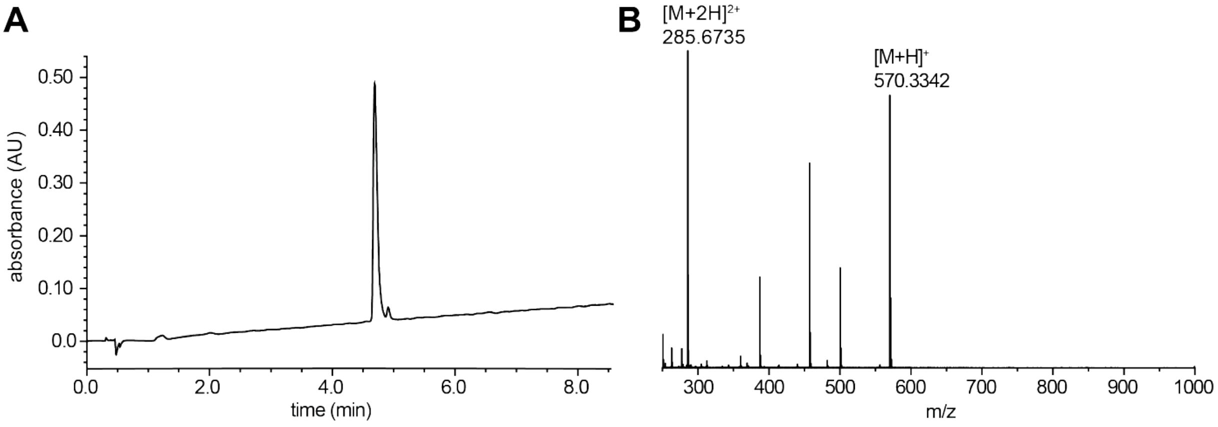

Figure S5. A) HPLC chromatogram (gradient from 0 to 25% ACN in water over 10 min at 2 ml/min, detection at 214 nm) and B) high resolution mass spectrum for peptide 5 LPRD-Sar.

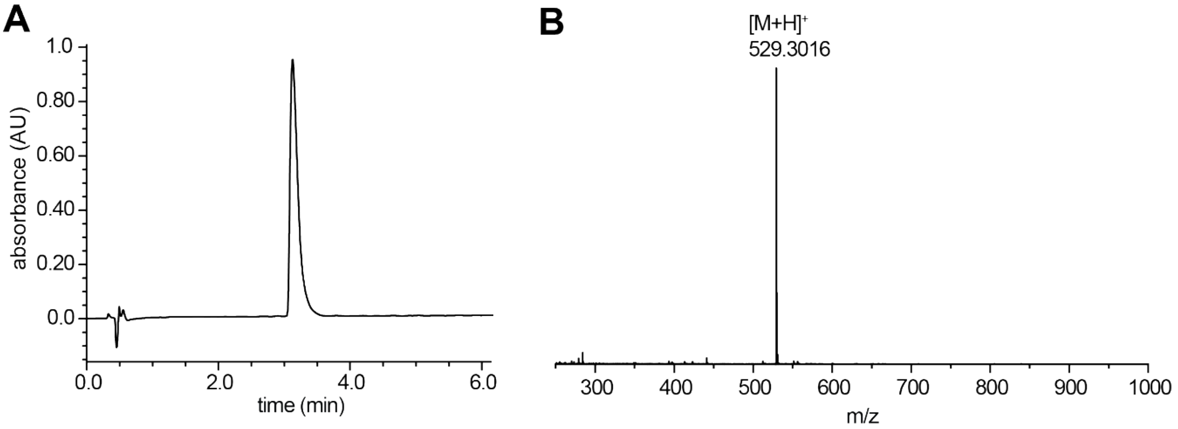

Figure S6. A) HPLC chromatogram (gradient from 5 to 25% ACN in water over 10 min at 2 ml/min, detection at 214 nm) and B) high resolution mass spectrum for peptide 6 LPET-Sar.

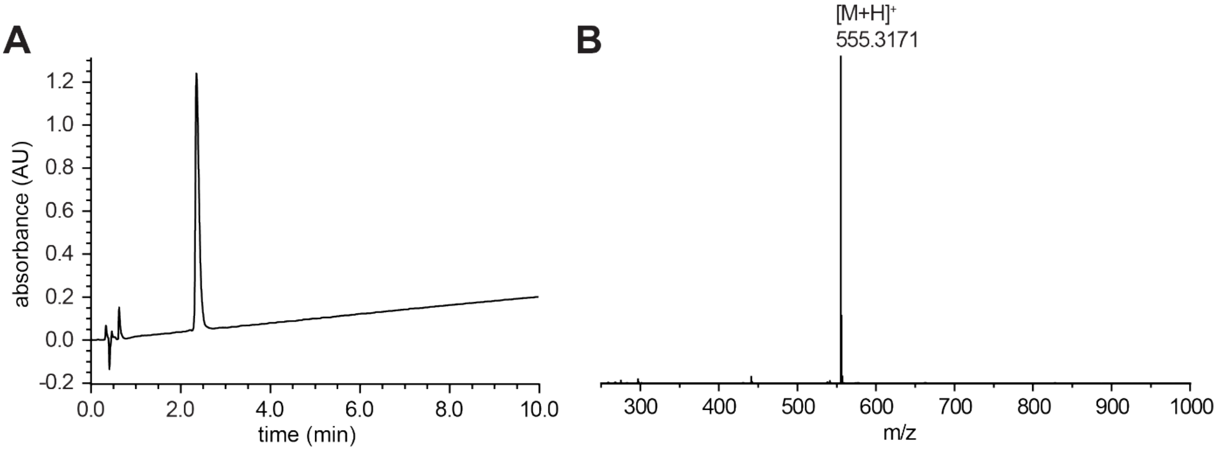

Figure S7. A) HPLC chromatogram (gradient from 5 to 50% ACN in water over 10 min at 2 ml/min, detection at 214 nm) and B) high resolution mass spectrum for peptide 7 LPETP.

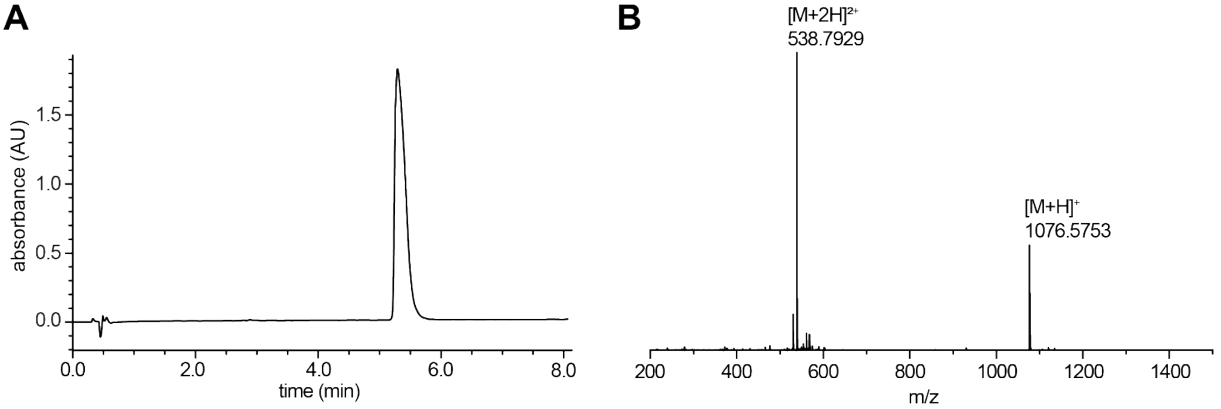

Figure S8. A) HPLC chromatogram (gradient from 5 to 35% ACN in water over 10 min at 2 ml/min, detection at 214 nm) and B) high resolution mass spectrum for peptide 8 KSFLPATGGAE.

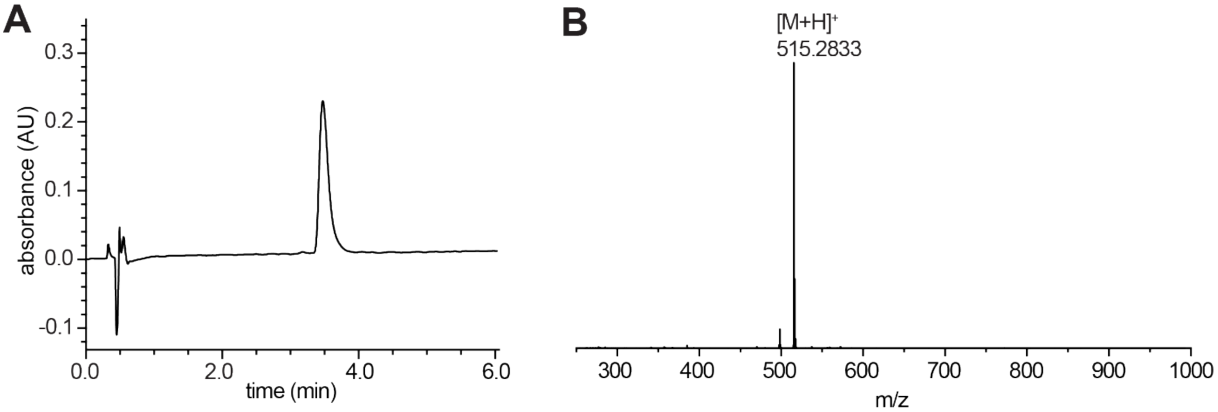

Figure S9. A) HPLC chromatogram (gradient from 5 to 25% ACN in water over 10 min at 2 ml/min, detection at 214 nm) and B) high resolution mass spectrum for peptide 9 GTEPL.

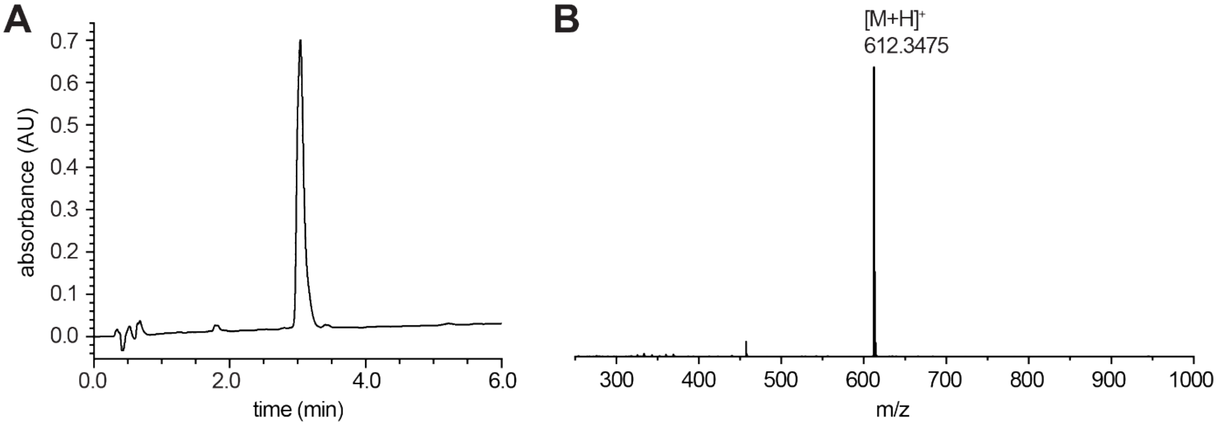

Figure S10. A) HPLC chromatogram (gradient from 5 to 50% ACN in water over 10 min at 2 ml/min, detection at 214 nm) and B) high resolution mass spectrum for peptide 10 AcLPRDA.

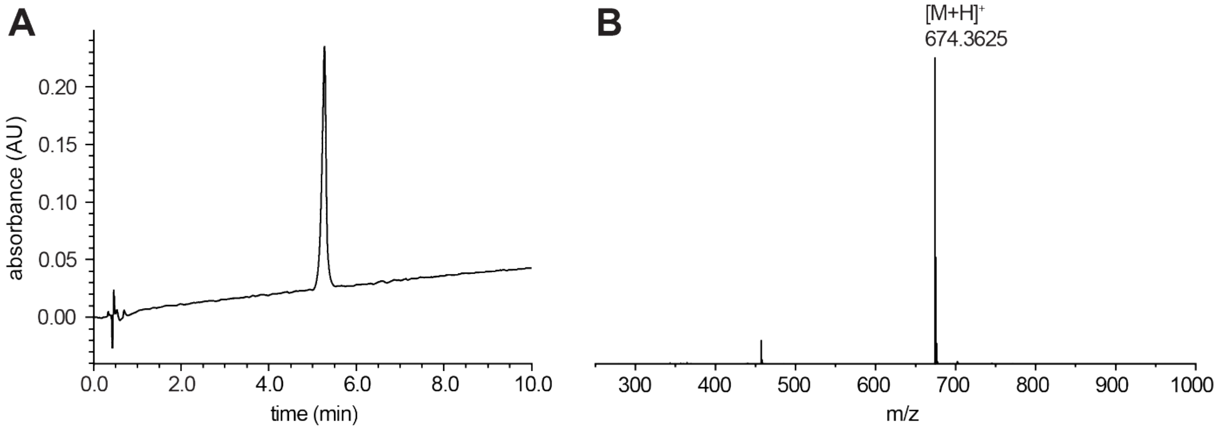

Figure S11. A) HPLC chromatogram (gradient from 5 to 50% ACN in water over 10 min at 2 ml/min, detection at 214 nm) and B) high resolution mass spectrum for peptide 11 BzLPRDA.

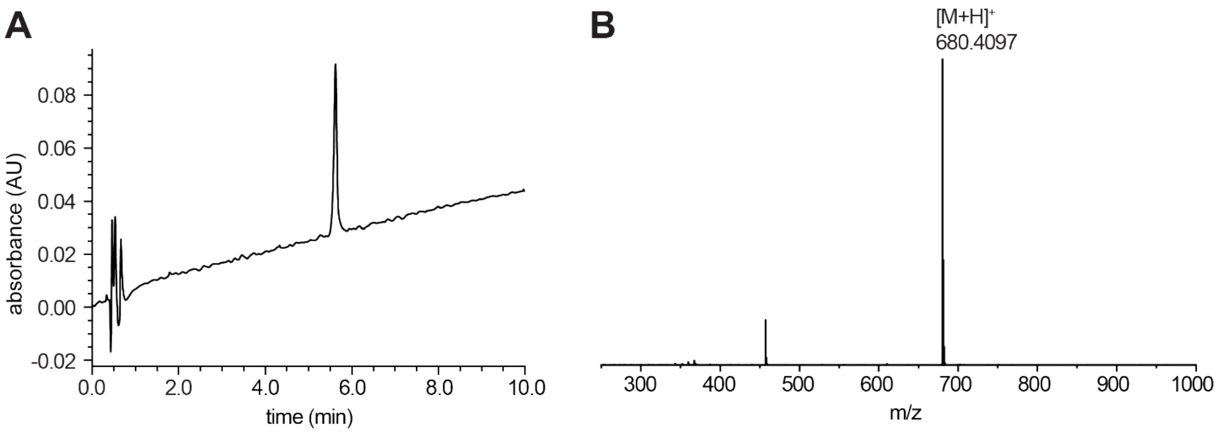

Figure S12. A) HPLC chromatogram (gradient from 5 to 50% ACN water over 10 min at 2 ml/min, detection at 214 nm) and B) high resolution mass spectrum for peptide 12 ChLPRDA.

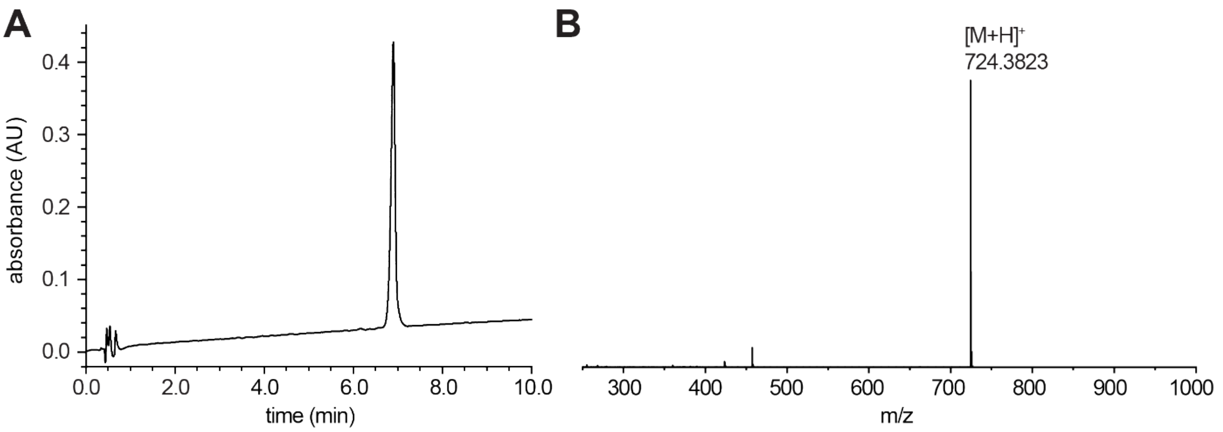

Figure S13. A) HPLC chromatogram (gradient from 5 to 50% ACN in water over 10 min at 2 ml/min, detection at 214 nm) and B) high resolution mass spectrum for peptide 13 2NapLPRDA.

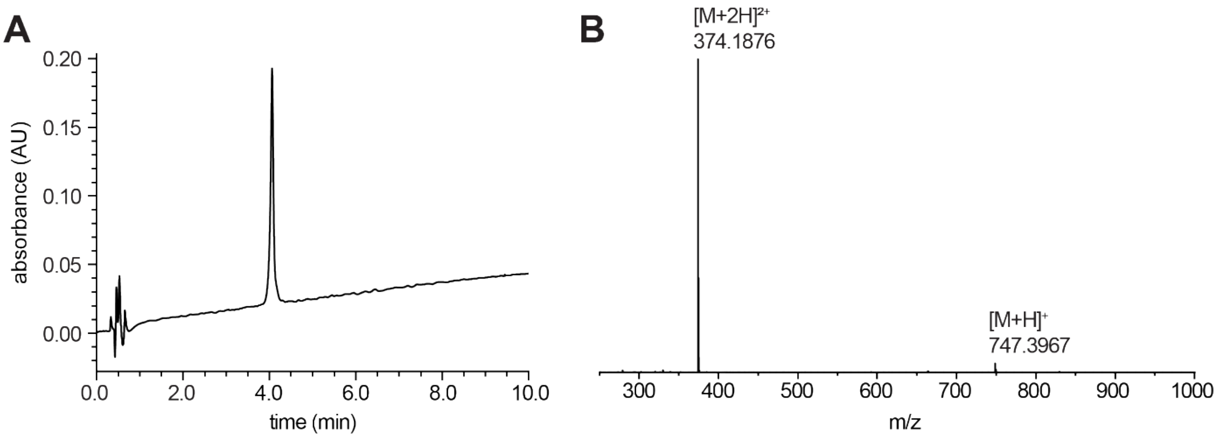

Figure S14. A) HPLC chromatogram (gradient from 5 to 50% ACN in water over 10 min at 2 ml/min, detection at 214 nm) and B) high resolution mass spectrum for peptide 14 CbzLPRDA.

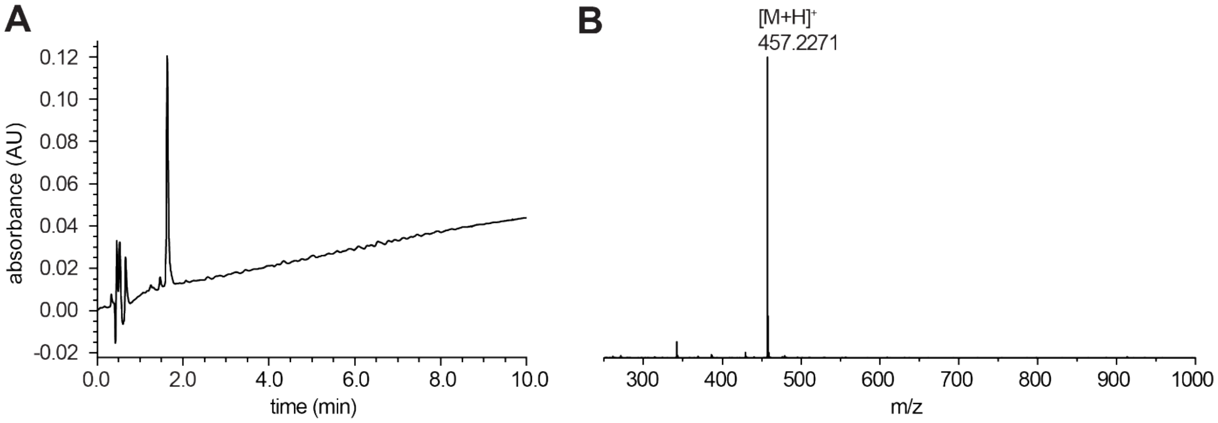

Figure S15. A) HPLC chromatogram (gradient from 5 to 50% ACN in water over 10 min at 2 ml/min, detection at 214 nm) and B) high resolution mass spectrum for peptide 15 AzARDA.

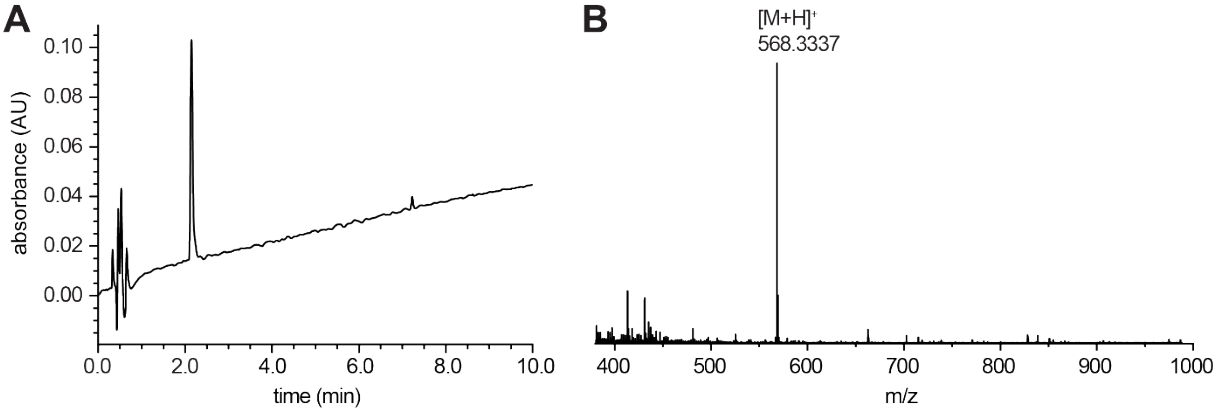

Figure S16. A) HPLC chromatogram (gradient from 5 to 50% ACN in water over 10 min at 2 ml/min, detection at 214 nm) and B) high resolution mass spectrum for peptide 16 LtARDA.

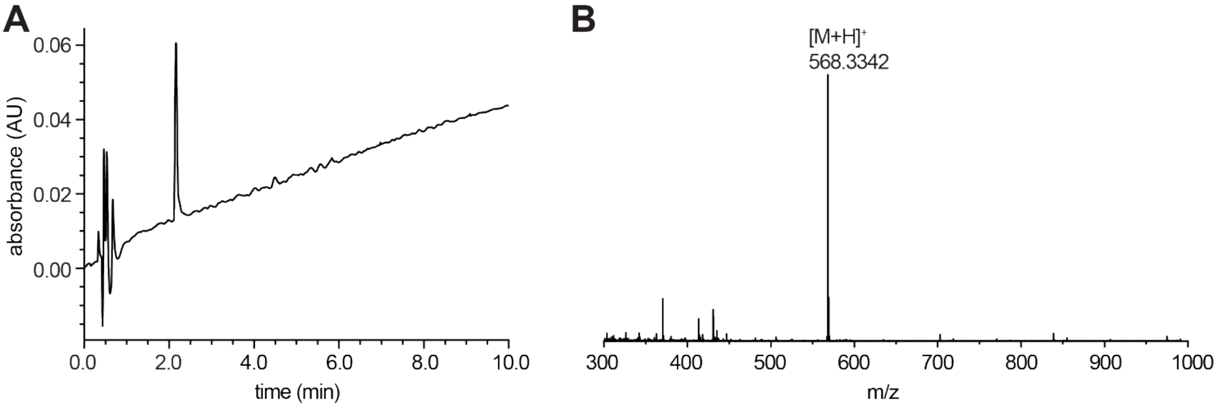

Figure S17. A) HPLC chromatogram (gradient from 5 to 50% ACN in water over 10 min at 2 ml/min, detection at 214 nm) and B) high resolution mass spectrum for peptide 17 LcARDA.

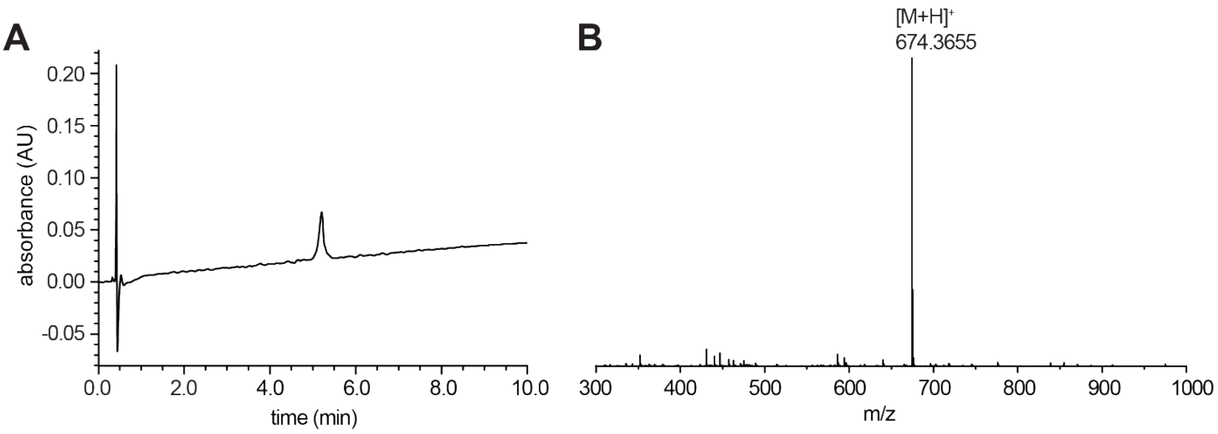

Figure S18. A) HPLC chromatogram (gradient from 5 to 50% ACN in water over 10 min at 2 ml/min, detection at 214 nm) and B) high resolution mass spectrum for peptide 18 BzLPRDSar.

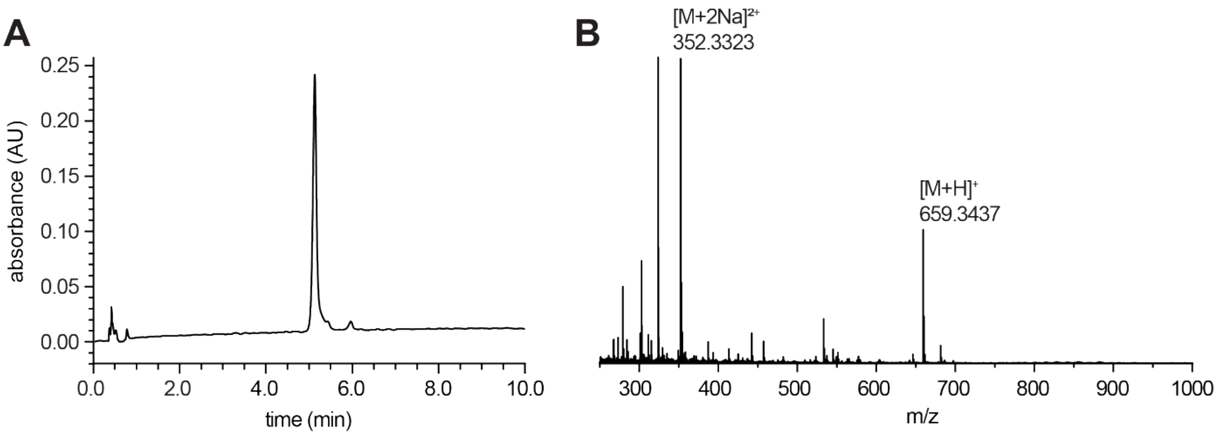

Figure S19. A) HPLC chromatogram (gradient from 5 to 50% ACN in water over 10 min at 2 ml/min, detection at 214 nm) and B) high resolution mass spectrum for peptide 19 BzLPETP.

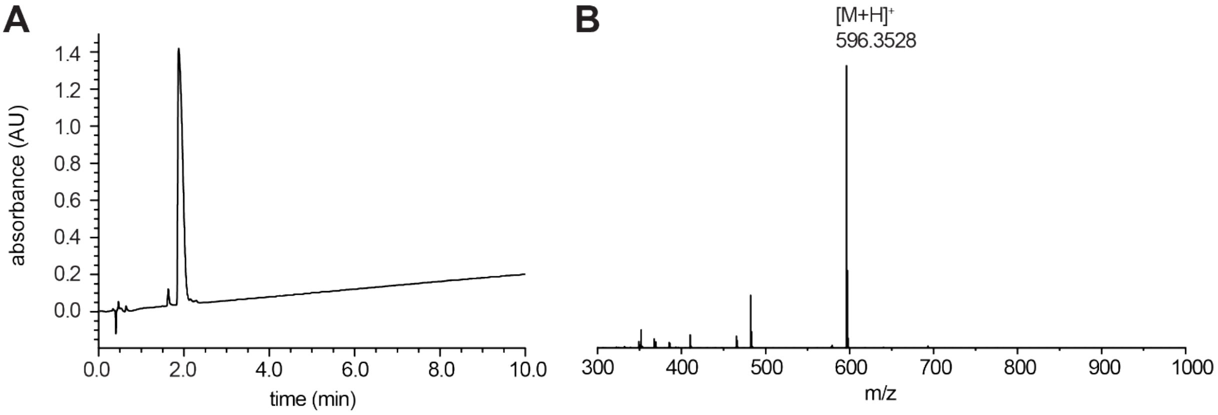

Figure S20. A) HPLC chromatogram (gradient from 5 to 50% ACN in water over 10 min at 2 ml/min, detection at 214 nm) and B) high resolution mass spectrum for peptide 20 LPRDP.

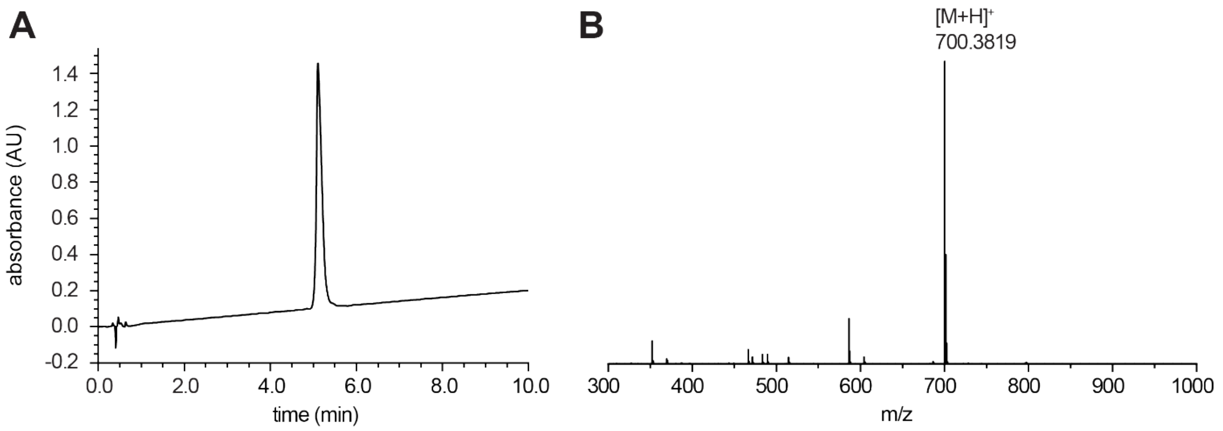

Figure S21. A) HPLC chromatogram (gradient from 5 to 50% ACN in water over 10 min at 2 ml/min, detection at 214 nm) and B) high resolution mass spectrum for peptide 21 BzLPRDP.

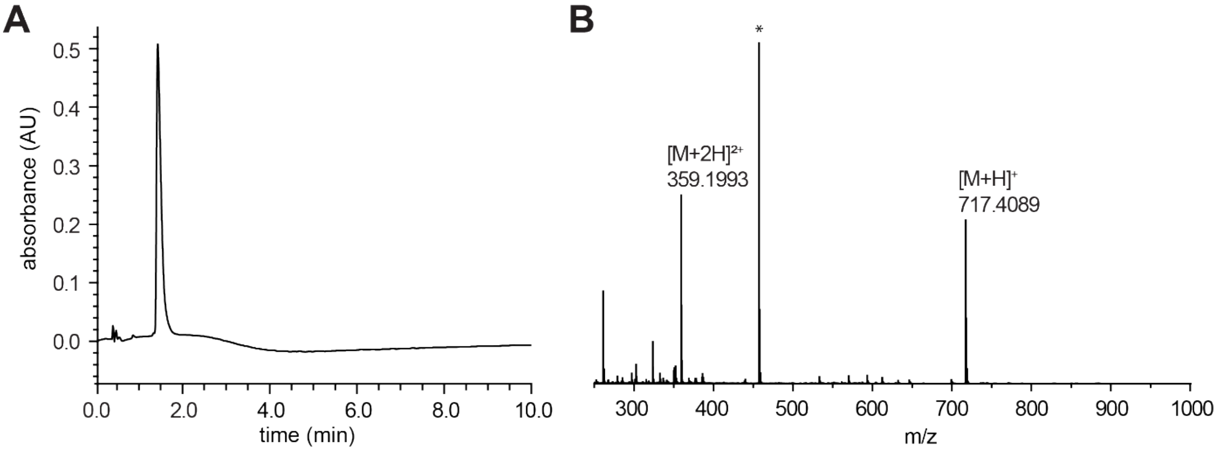

Figure S22. A) HPLC chromatogram (gradient from 5 to 50% ACN in water over 10 min at 2 ml/min, detection at 214 nm) and B) high resolution mass spectrum for peptide 22 FLPRDA. * impurity in the LC-MS system.

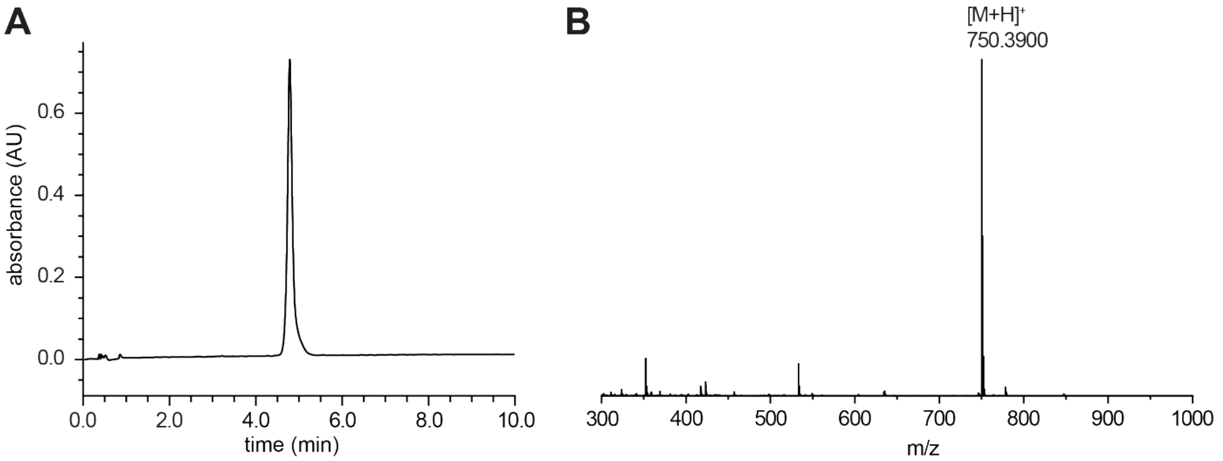

Figure S23. A) HPLC chromatogram (gradient from 5 to 50% ACN in water over 10 min at 2 ml/min, detection at 214 nm) and B) high resolution mass spectrum for peptide 23 BzLPRDF.

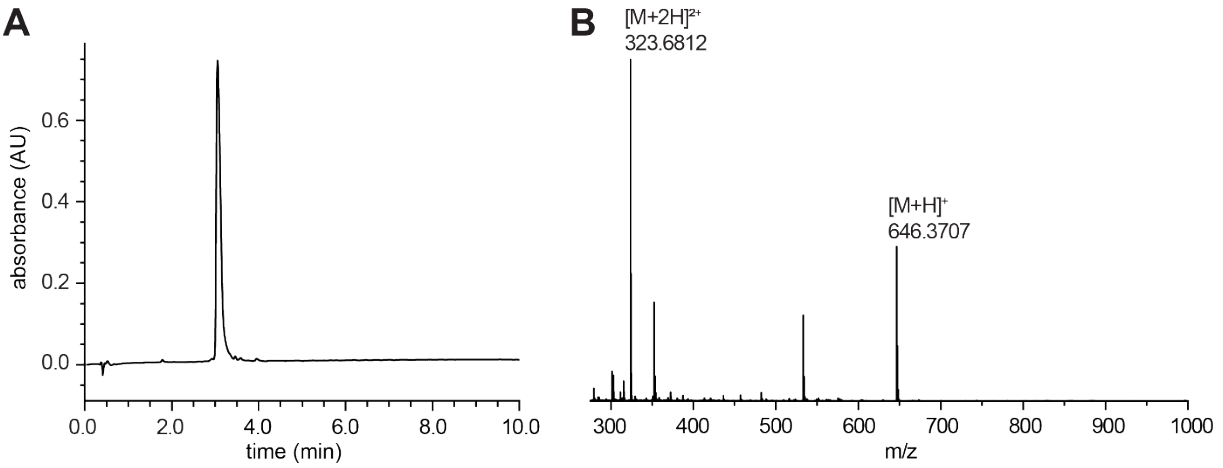

Figure S24. A) HPLC chromatogram (gradient from 5 to 50% ACN in water over 10 min at 2 ml/min, detection at 214 nm) and B) high resolution mass spectrum for peptide 24 LPRDF.

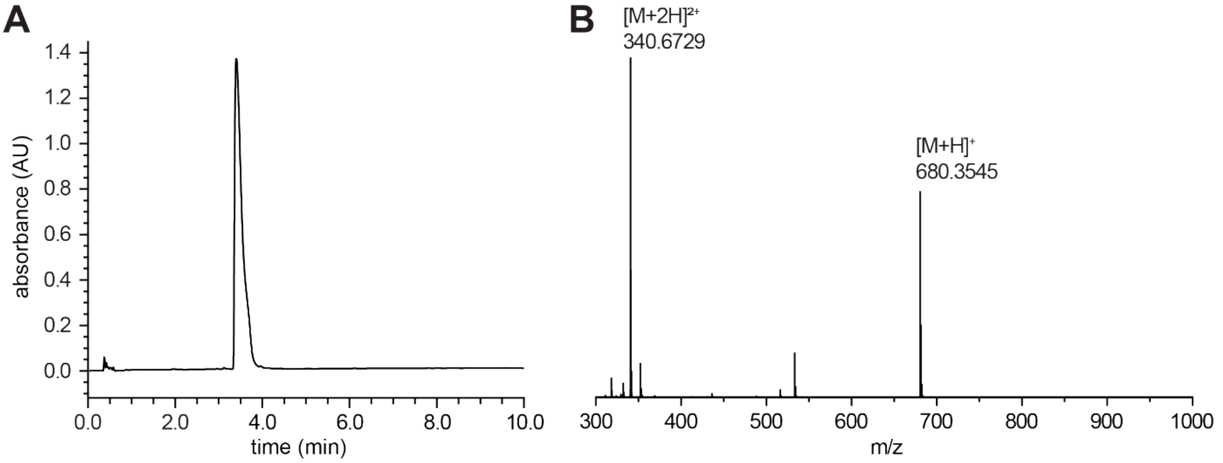

Figure S25. A) HPLC chromatogram (gradient from 5 to 50% ACN in water over 10 min at 2 ml/min, detection at 214 nm) and B) high resolution mass spectrum for peptide 25 FPRDF.

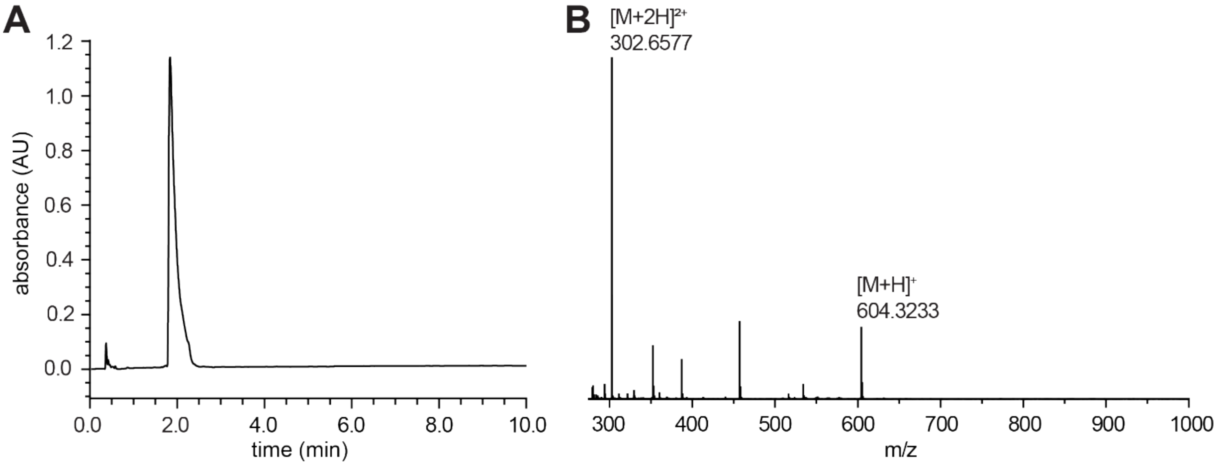

Figure S26. A) HPLC chromatogram (gradient from 5 to 50% ACN in water over 10 min at 2 ml/min, detection at 214 nm) and B) high resolution mass spectrum for peptide 26 FPRDPSar.

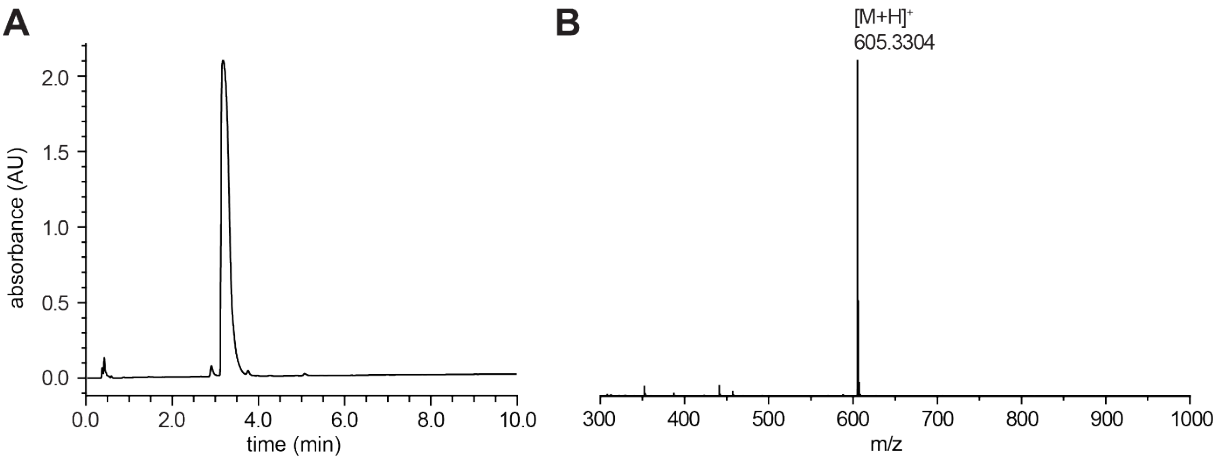

Figure S27. A) HPLC chromatogram (gradient from 5 to 50% ACN in water over 10 min at 2 ml/min, detection at 214 nm) and B) high resolution mass spectrum for peptide 27 LPETF.

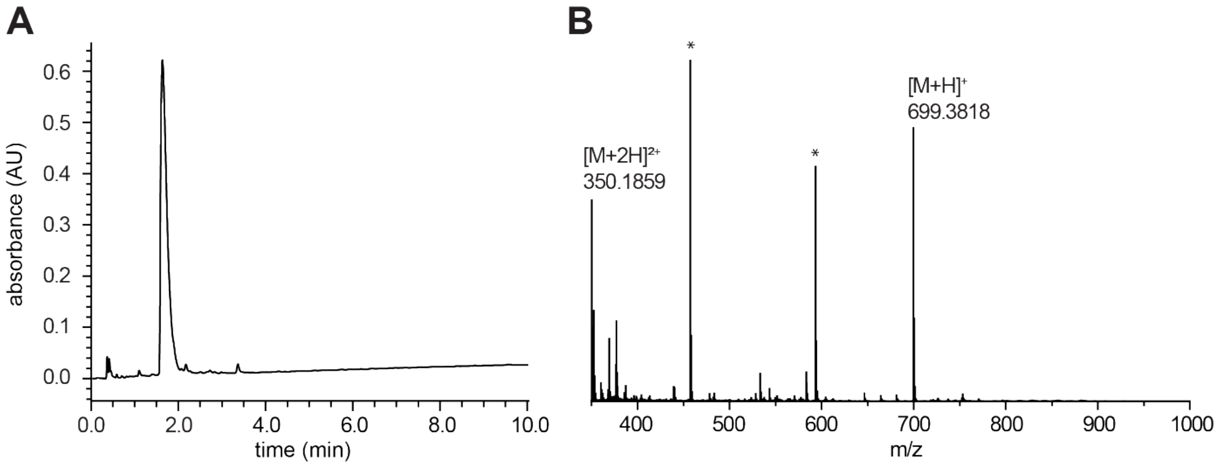

Figure S28. A) HPLC chromatogram (gradient from 5 to 50% ACN in water over 10 min at 2 ml/min, detection at 214 nm) and B) high resolution mass spectrum for peptide 28 ELPRDA. * impurities from the LC-MS system

Sortase A enzyme expression and purification

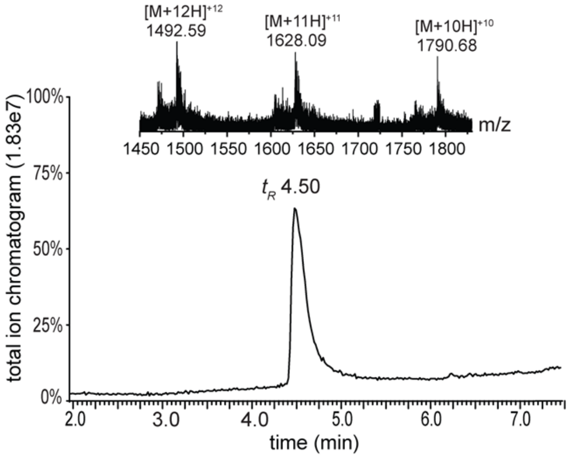

Figure S29. LC-MS spectra for purified SrtA protein. RP-HPLC elution profile (lower panel; gradient from 5 to 95% of acetonitrile containing 0.1% formic acid (F.A) over 10 min at a flow rate of 0.3 ml/min and detection at 214 nm) and ESI-MS (upper panel) spectrum of the protein showing $[M+10H]^{10+}$, $[M+11H]^{11+}$ and $[M+12H]^{12+}$ fragments of SrtA. LC-MS calculated $C_{787}H_{1238}O_{241}N_{220}S_4$ 17766.01; observed m/z 1492.59 $[M+12H]^{+12}$

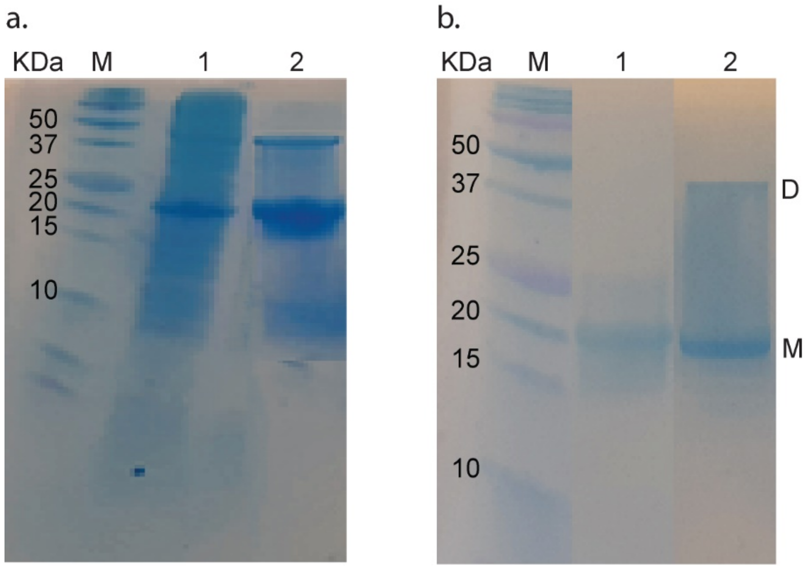

Figure S30. SDS-PAGE analysis for the expressed Sortase A enzyme. **a.** SDS-PAGE analysis after cell expression and following IMAC. Lane M: Protein marker. Lane 1: Supernatant from E. coli BL21 transfected with pET21b plasmid before purification. Lane 2: Supernatant from E. coli BL21 transfected with pET21b plasmid after purification using Ni Sepharose 6 Fast Flow columns. The estimated molecular weight of SrtA protein was approximately 20 kDa as indicated by SDS-PAGE. **b.** SDS-PAGE analysis following FPLC purification. Lane M: Protein marker. Lane 1: Monomer of SrtA protein under reducing gel conditions without dimer band. Lane 2: Dimer and monomer of SrtA protein. The band labelled with D indicates the band of the dimeric protein and the band labelled with M indicates the band of monomeric protein.

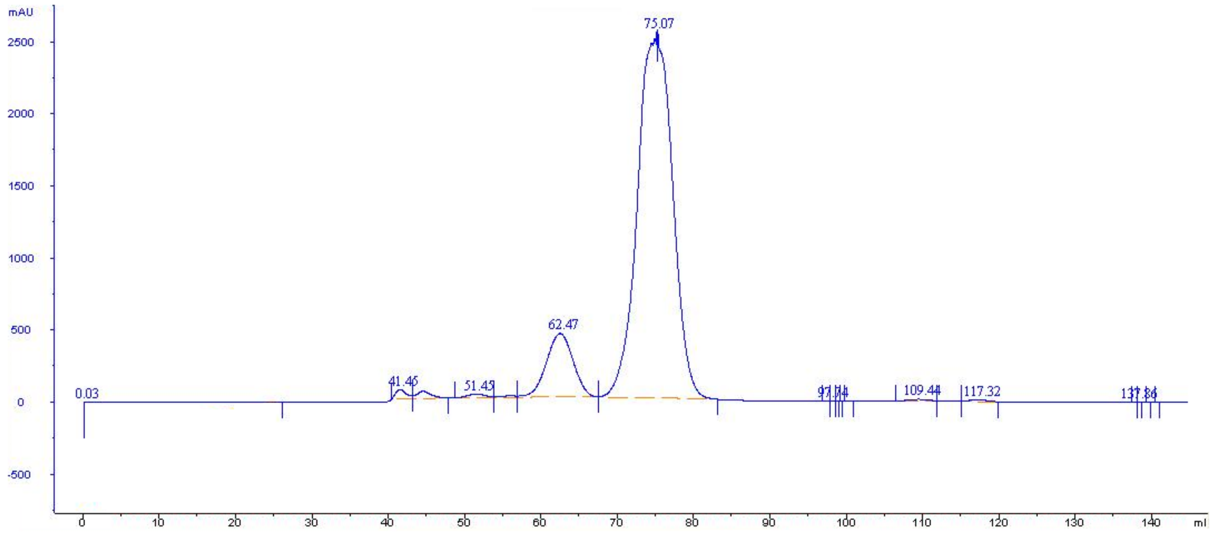

Figure S31. Fast protein liquid chromatography (FPLC) profile of SrtA protein purification.

Biochemical assays

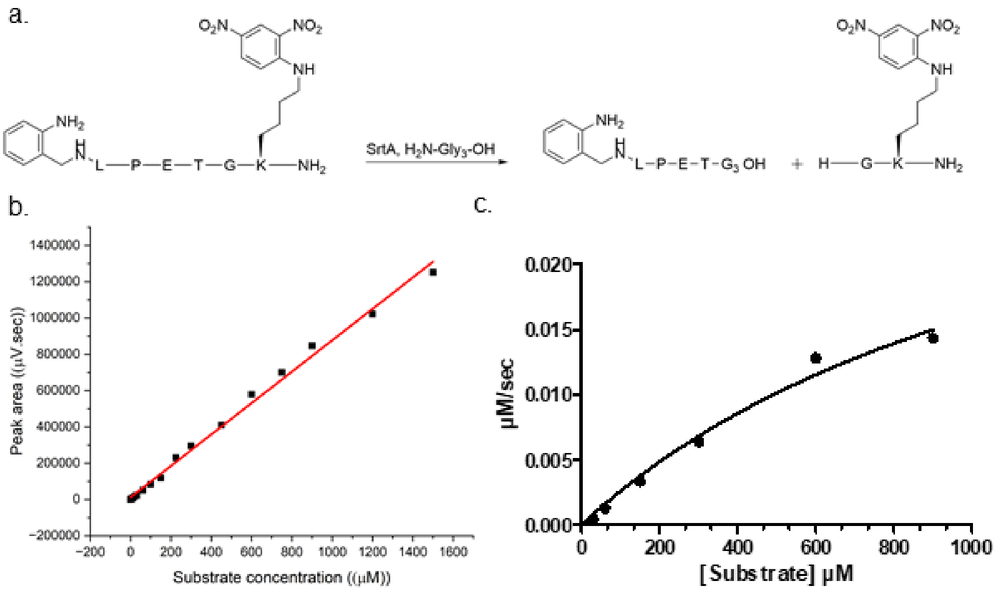

Figure S32. a. Sortase-catalyzed transpeptidation reaction of Abz-LPETGK(Dnp)-NH_2 substrate and triglycine; **b.** Calibration curve for the kinetic assay, correlating the peak area measured in HPLC with substrate concentration for Abz-LPETGK(Dnp)-NH_2; **c.** RP-HPLC-based SrtA kinetic assay showing the Michealis-Menten plot. Reaction velocities were calculated from the change of peak area over time and converted to concentration as described in the experimental procedures.

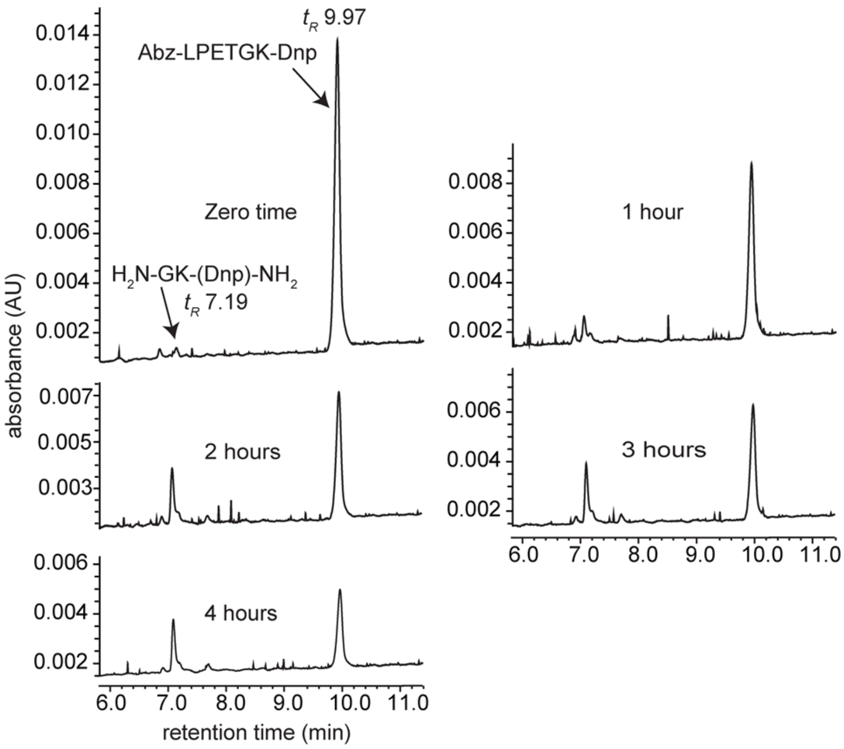

Figure S33. RP-HPLC elution profile of SrtA catalyzed cleavage of the fluorogenic substrate Abz-LPETGK(Dnp)-NH_2 over 4 hrs.

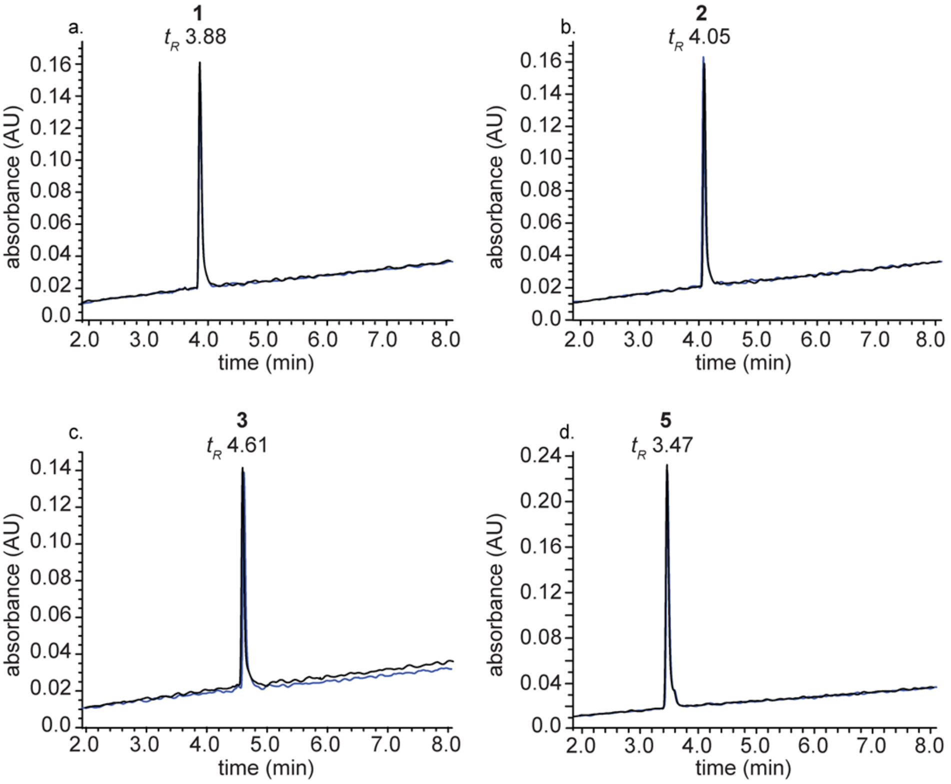

Figure S34. RP-HPLC monitoring of SrtA catalyzed cleavage of the synthetic peptides **a.** H2N-LPRDA-OH (1), **b.** LPRDA-Ado (2), **c.** Ado-LPRDA (3), and **d.** LPRD-Sar (5). The blue HPLC traces correspond to the analysis before incubation with the enzyme and the black traces correspond to the analysis after 4 hrs of incubation with the enzyme.

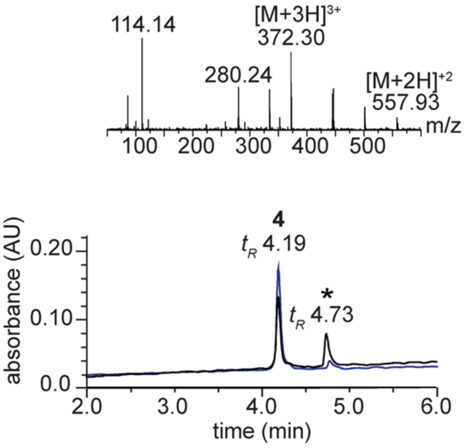

Figure S35. RP-HPLC and LC-MS monitoring of SrtA catalyzed cleavage of the synthetic peptide **4**. The blue HPLC traces correspond to the analysis before incubation with the enzyme and the black traces correspond to the analysis after 4 hrs of incubation with the enzyme (lower panel). The asterisks (*) correspond to the peaks collected from RP-HPLC and verified by ESI-MS spectrometer (upper panel).

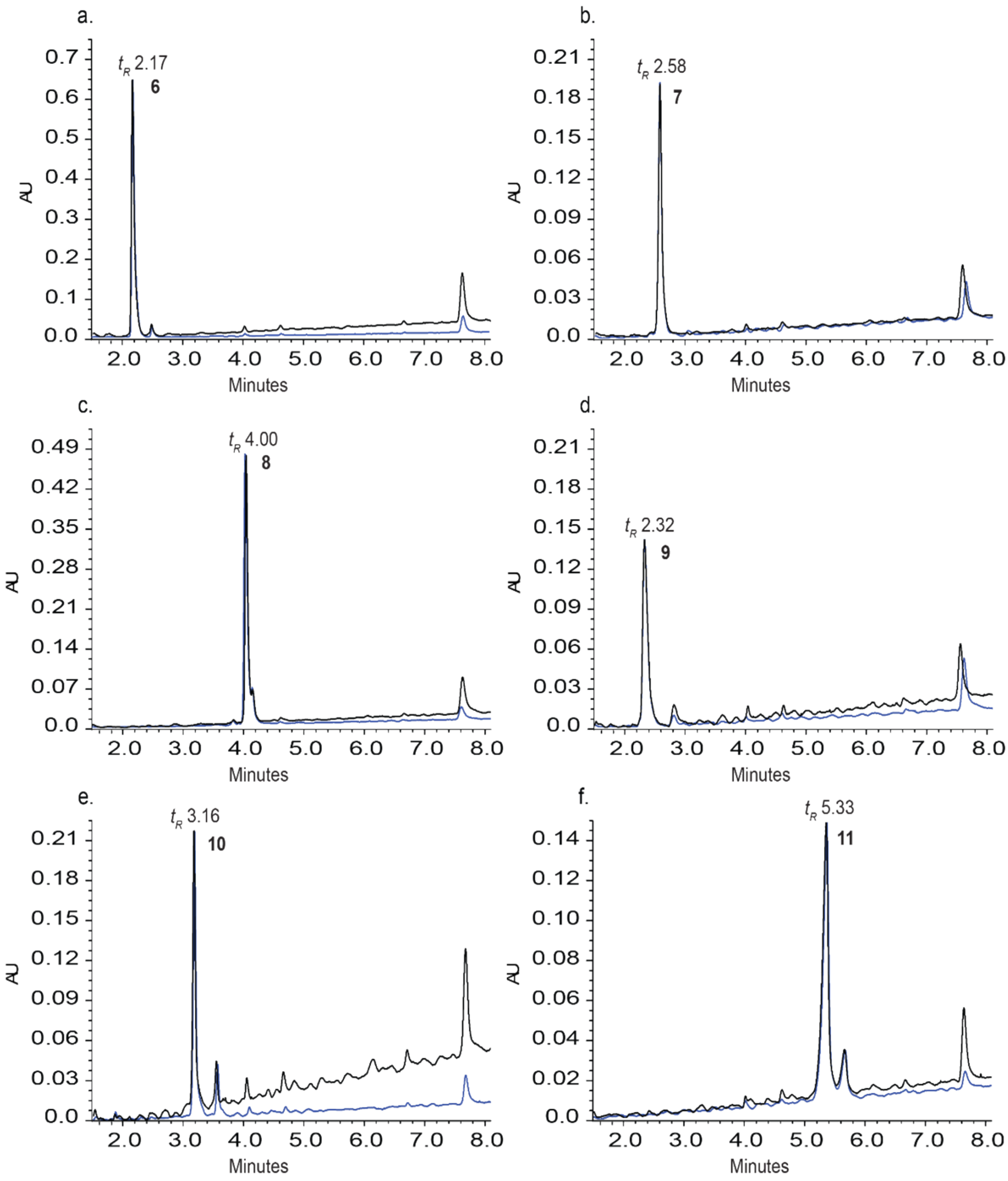

Figure S36 (continues). RP-HPLC monitoring of SrtA catalyzed cleavage of the synthetic peptides **a.** LPET-Sar (6), **b.** LPETP (7), **c.** KSFLPATGGAE (8), **d.** GTEPL (9), **e.** AcLPRDA (10), **f.** BzLPRDA (11), **g.** ChLPRDA (12), **h.** 2NapLPRDA (13), **i.** CbzLPRDA (14), **j.** AzARDA (15), **k.** LtARDA (16), **l.** LcARDA (17), **m)** BzLPRDSar (18), **n.** BzLPETP (19), **o.** LPRDP (20), **p.** BzLPRDP (21), **q.** FLPRDA (22), **r.** BzLPRDF (23), **s.** LPRDF (24), **t.** FPRDF (25), **u.** FPRDSar (26), **v.** LPETF (27) and **w.** ELPRDA (28). The blue HPLC traces correspond to the analysis before incubation with the enzyme and the black traces correspond to the analysis after 4 hrs of incubation with the enzyme.

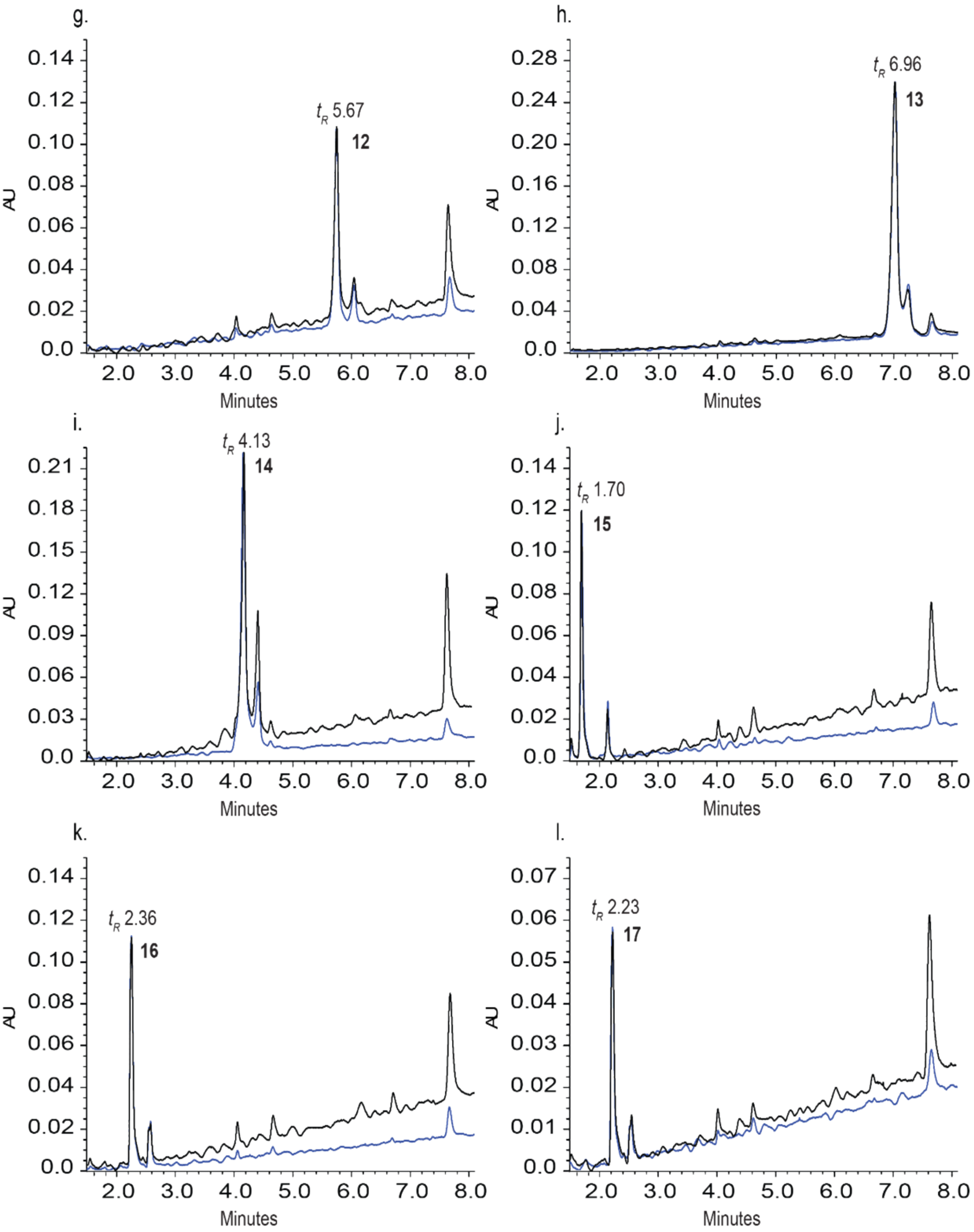

Figure S36 (continues). RP-HPLC monitoring of SrtA catalyzed cleavage of the synthetic peptides **a.** LPET-Sar (6), **b.** LPETP (7), **c.** KSFLPATGGAE (8), **d.** GTEPL (9), **e.** AcLPRDA (10), **f.** BzLPRDA (11), **g.** ChLPRDA (12), **h.** 2NapLPRDA (13), **i.** CbzLPRDA (14), **j.** AzARDA (15), **k.** LtARDA (16), **l.** LcARDA (17), **m)** BzLPRDSar (18), **n.** BzLPETP (19), **o.** LPRDP (20), **p.** BzLPRDP (21), **q.** FLPRDA (22), **r.** BzLPRDF (23), **s.** LPRDF (24), **t.** FPRDF (25), **u.** FPRDSar (26), **v.** LPETF (27) and **w.** ELPRDA (28). The blue HPLC traces correspond to the analysis before incubation with the enzyme and the black traces correspond to the analysis after 4 hrs of incubation with the enzyme.

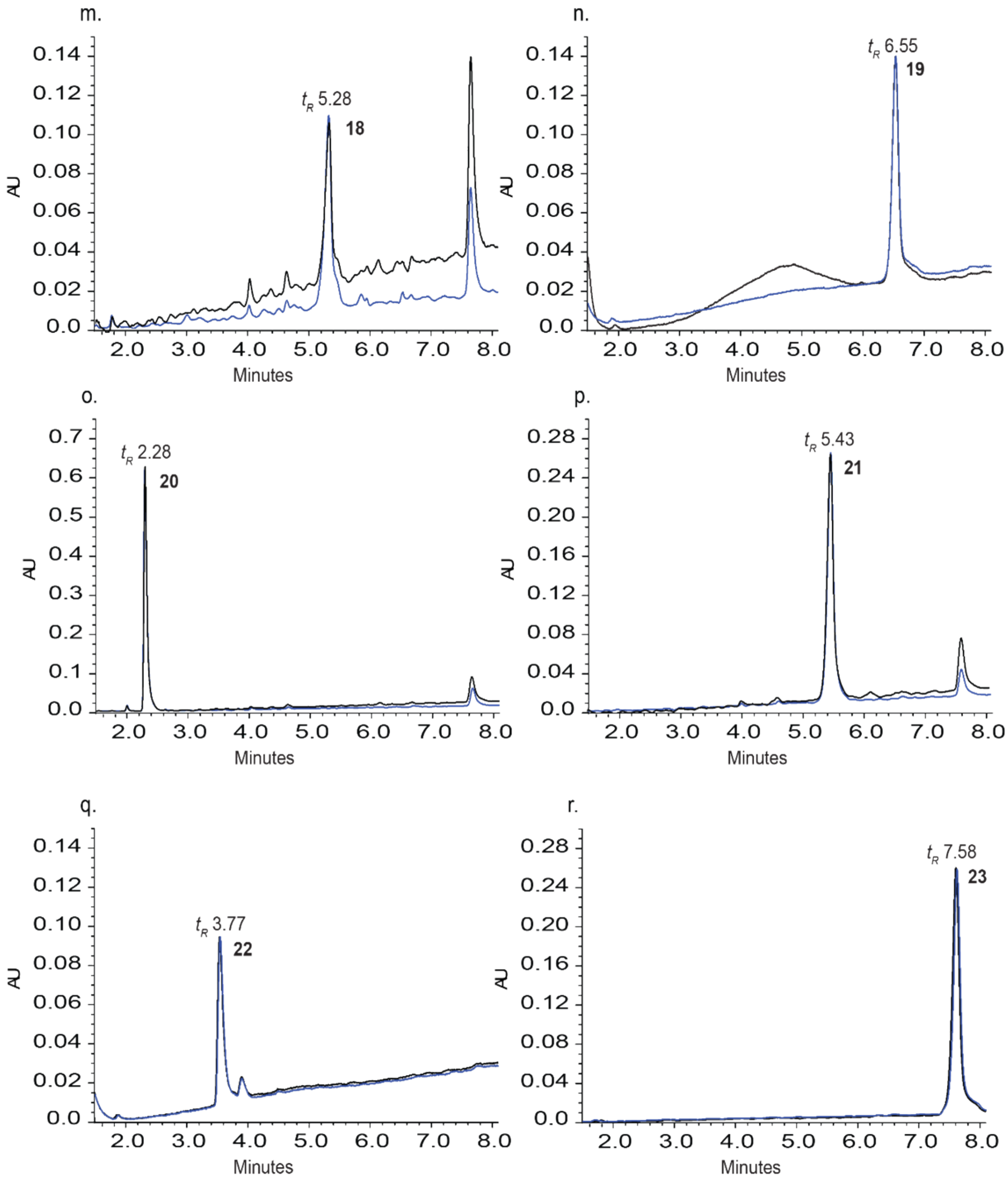

Figure S36 (continues). RP-HPLC monitoring of SrtA catalyzed cleavage of the synthetic peptides **a.** LPET-Sar (6), **b.** LPETP (7), **c.** KSFLPATGGAE (8), **d.** GTEPL (9), **e.** AcLPRDA (10), **f.** BzLPRDA (11), **g.** ChLPRDA (12), **h.** 2NapLPRDA (13), **i.** CbzLPRDA (14), **j.** AzARDA (15), **k.** LtARDA (16), **l.** LcARDA (17), **m)** BzLPRDSar (18), **n.** BzLPETP (19), **o.** LPRDP (20), **p.** BzLPRDP (21), **q.** FLPRDA (22), **r.** BzLPRDF (23), **s.** LPRDF (24), **t.** FPRDF (25), **u.** FPRDSar (26), **v.** LPETF (27) and **w.** ELPRDA (28). The blue HPLC traces correspond to the analysis before incubation with the enzyme and the black traces correspond to the analysis after 4 hrs of incubation with the enzyme.

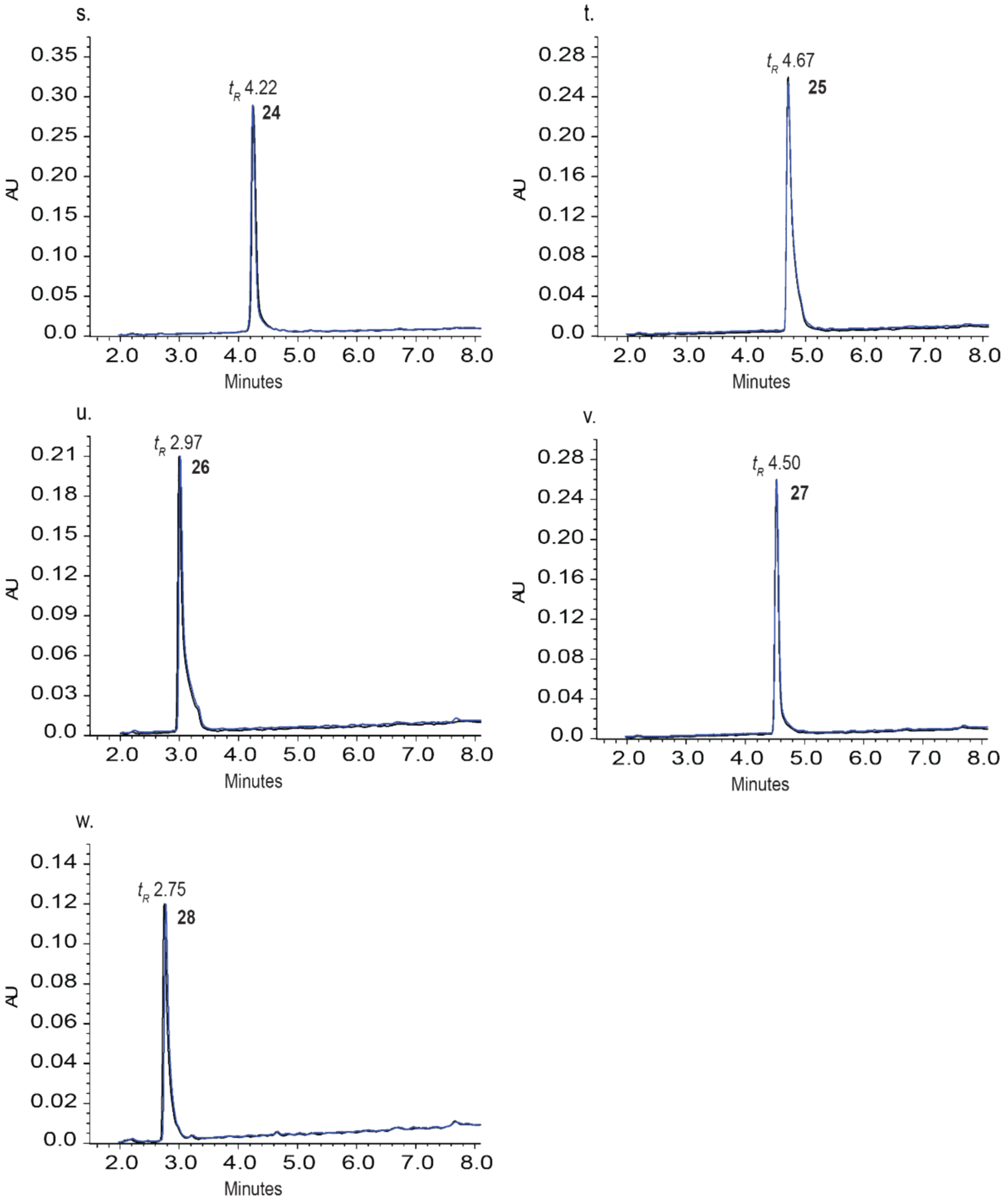

Figure S36. RP-HPLC monitoring of SrtA catalyzed cleavage of the synthetic peptides **a.** LPET-Sar (6), **b.** LPETP (7), **c.** KSFLPATGGAE (8), **d.** GTEPL (9), **e.** AcLPRDA (10), **f.** BzLPRDA (11), **g.** ChLPRDA (12), **h.** 2NapLPRDA (13), **i.** CbzLPRDA (14), **j.** AzARDA (15), **k.** LtARDA (16), **l.** LcARDA (17), **m)** BzLPRDSar (18), **n.** BzLPETP (19), **o.** LPRDP (20), **p.** BzLPRDP (21), **q.** FLPRDA (22), **r.** BzLPRDF (23), **s.** LPRDF (24), **t.** FPRDF (25), **u.** FPRDSar (26), **v.** LPETF (27) and **w.** ELPRDA (28). The blue HPLC traces correspond to the analysis before incubation with the enzyme and the black traces correspond to the analysis after 4 hrs of incubation with the enzyme.

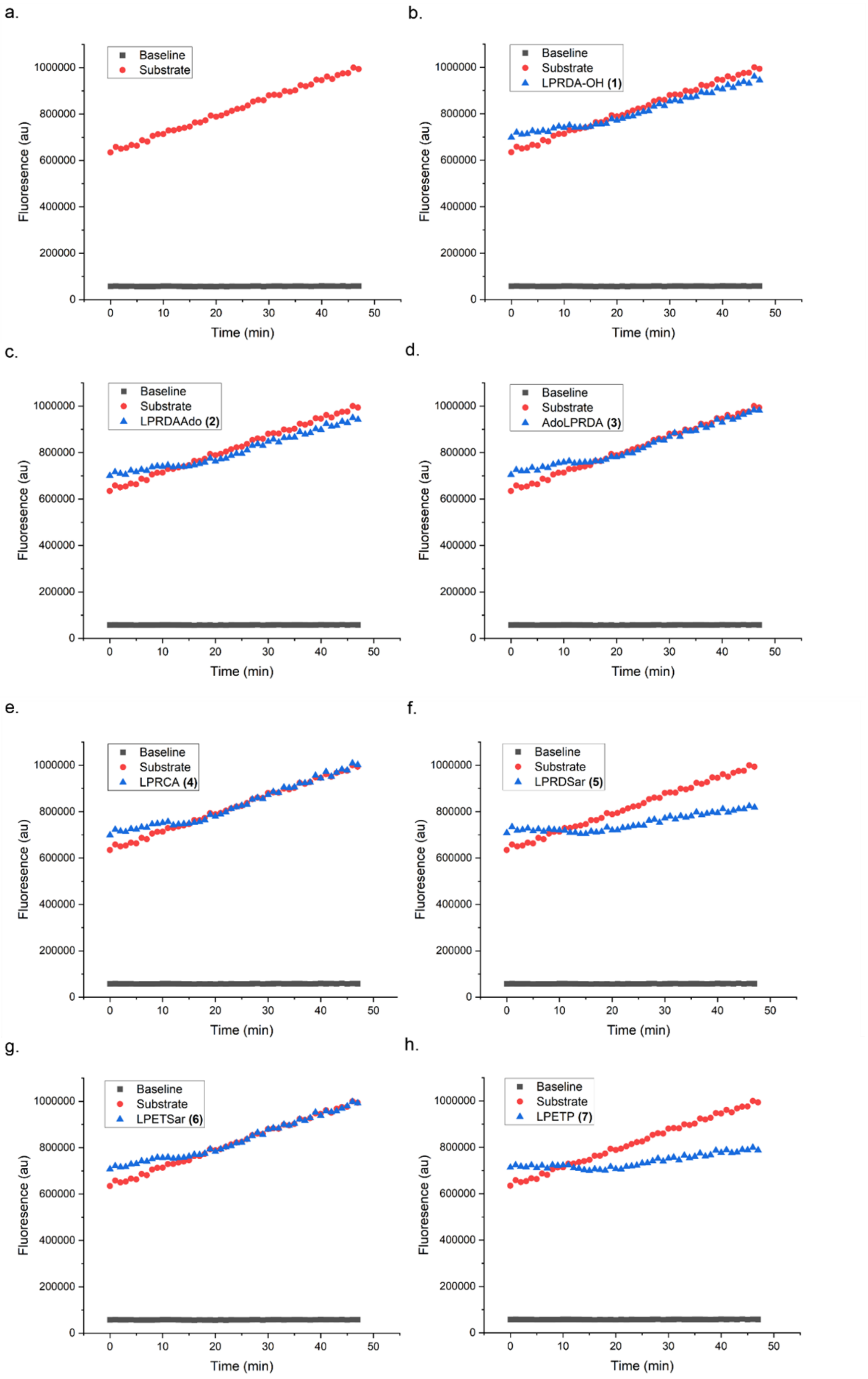

Figure S37 (continues). FRET-based assay results for the tested peptidomimetic compounds represented as fluorescence signal readouts plotted against time over 45 min for each compound. **A.** Abz-LPETGK(Dnp)-NH^{2} Substrate, **b.** 1, **c.** 2, **d.** 3, **e.** 4, **f.** 5, **g.** 6, **h.** 7, **i.** 8, **j.** 9, **k.** 10, **l.** 11, **m.** 12, **n.** 13, **o.** 14, **p.** 15, **q.** 16, **r.** 17, **s.** 18, **t.** 19, **u.** 20, **v.** 21, **w.** 22, **x.** 23, **y.** 24, **z.** 25, **aa.** 26, **ab.** 27, **ac.** 28 and **ad.** reference. Each inhibitor was assayed in triplicate and average values were determined.

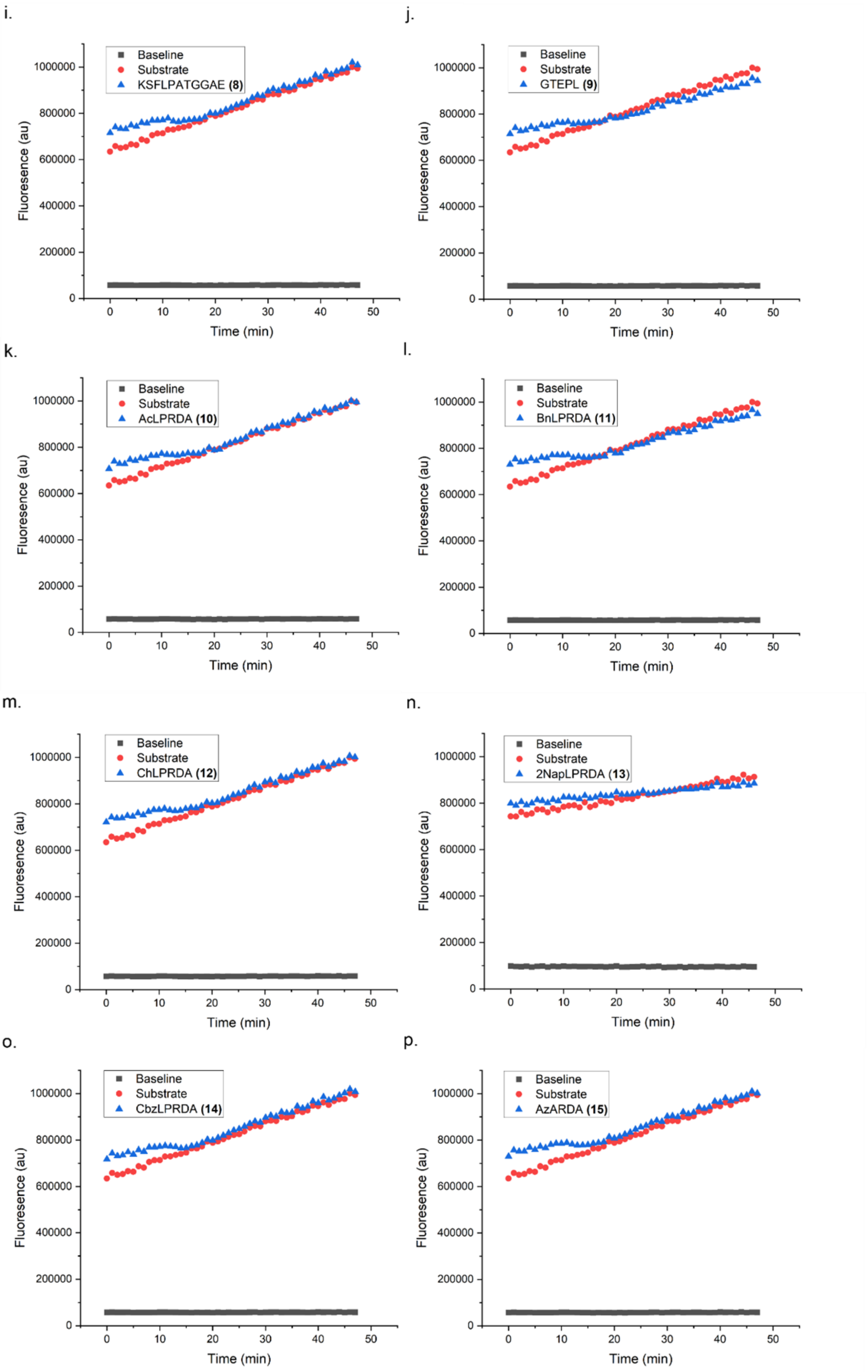

Figure S37 (continues). FRET-based assay results for the tested peptidomimetic compounds represented as fluorescence signal readouts plotted against time over 45 min for each compound. **A.** Abz-LPETGK(Dnp)-NH^2 Substrate, **b.** 1, **c.** 2, **d.** 3, **e.** 4, **f.** 5, **g.** 6, **h.** 7, **i.** 8, **j.** 9, **k.** 10, **l.** 11, **m.** 12, **n.** 13, **o.** 14, **p.** 15, **q.** 16, **r.** 17, **s.** 18, **t.** 19, **u.** 20, **v.** 21, **w.** 22, **x.** 23, **y.** 24, **z.** 25, **aa.** 26, **ab.** 27, **ac.** 28 and **ad.** reference. Each inhibitor was assayed in triplicate and average values were determined.

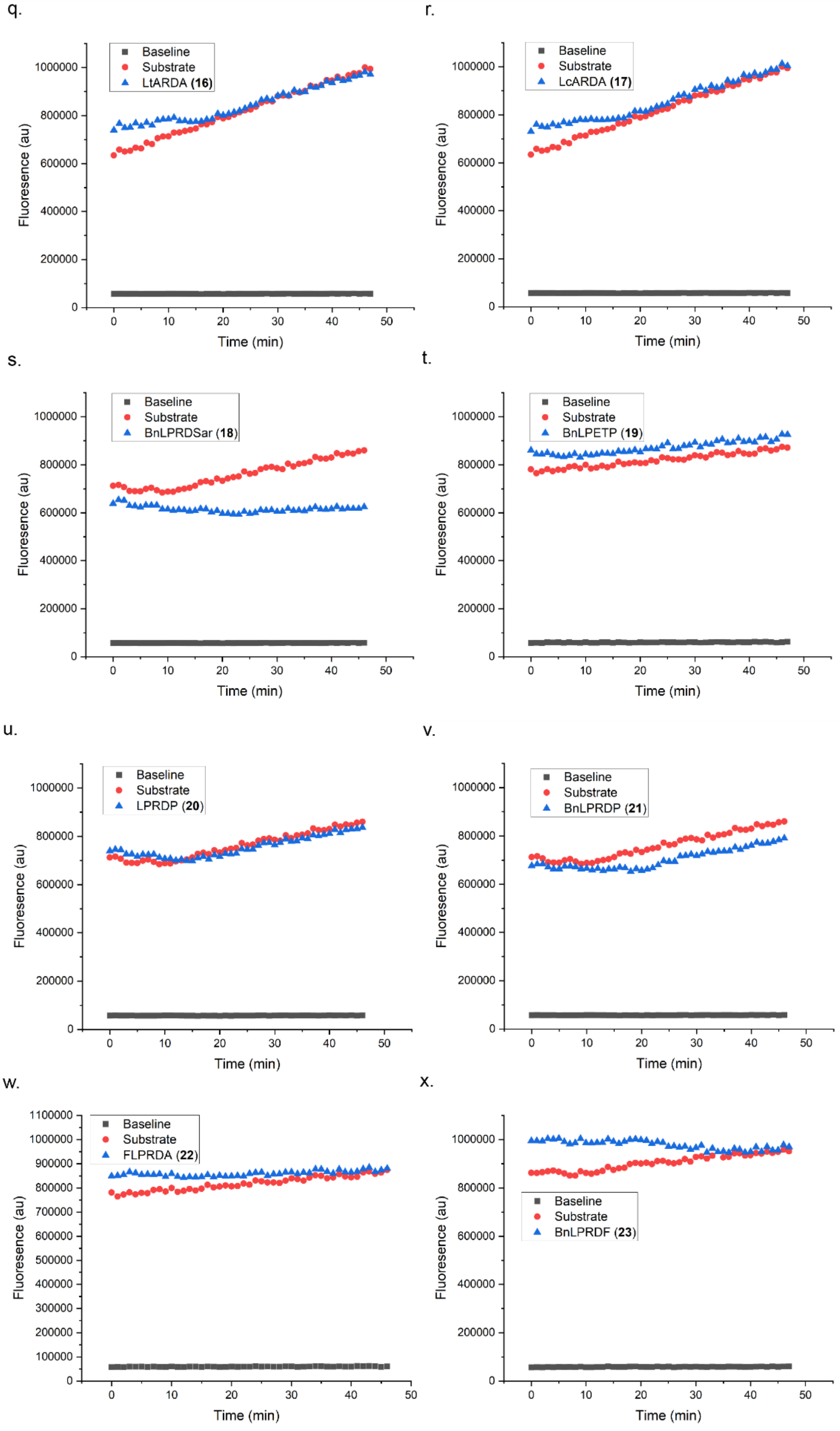

Figure S37 (continues). FRET-based assay results for the tested peptidomimetic compounds represented as fluorescence signal readouts plotted against time over 45 min for each compound. **A.** Abz-LPETGK(Dnp)-NH^2 Substrate, **b.** 1, **c.** 2, **d.** 3, **e.** 4, **f.** 5, **g.** 6, **h.** 7, **i.** 8, **j.** 9, **k.** 10, **l.** 11, **m.** 12, **n.** 13, **o.** 14, **p.** 15, **q.** 16, **r.** 17, **s.** 18, **t.** 19, **u.** 20, **v.** 21, **w.** 22, **x.** 23, **y.** 24, **z.** 25, **aa.** 26, **ab.** 27, **ac.** 28 and **ad.** reference. Each inhibitor was assayed in triplicate and average values were determined.

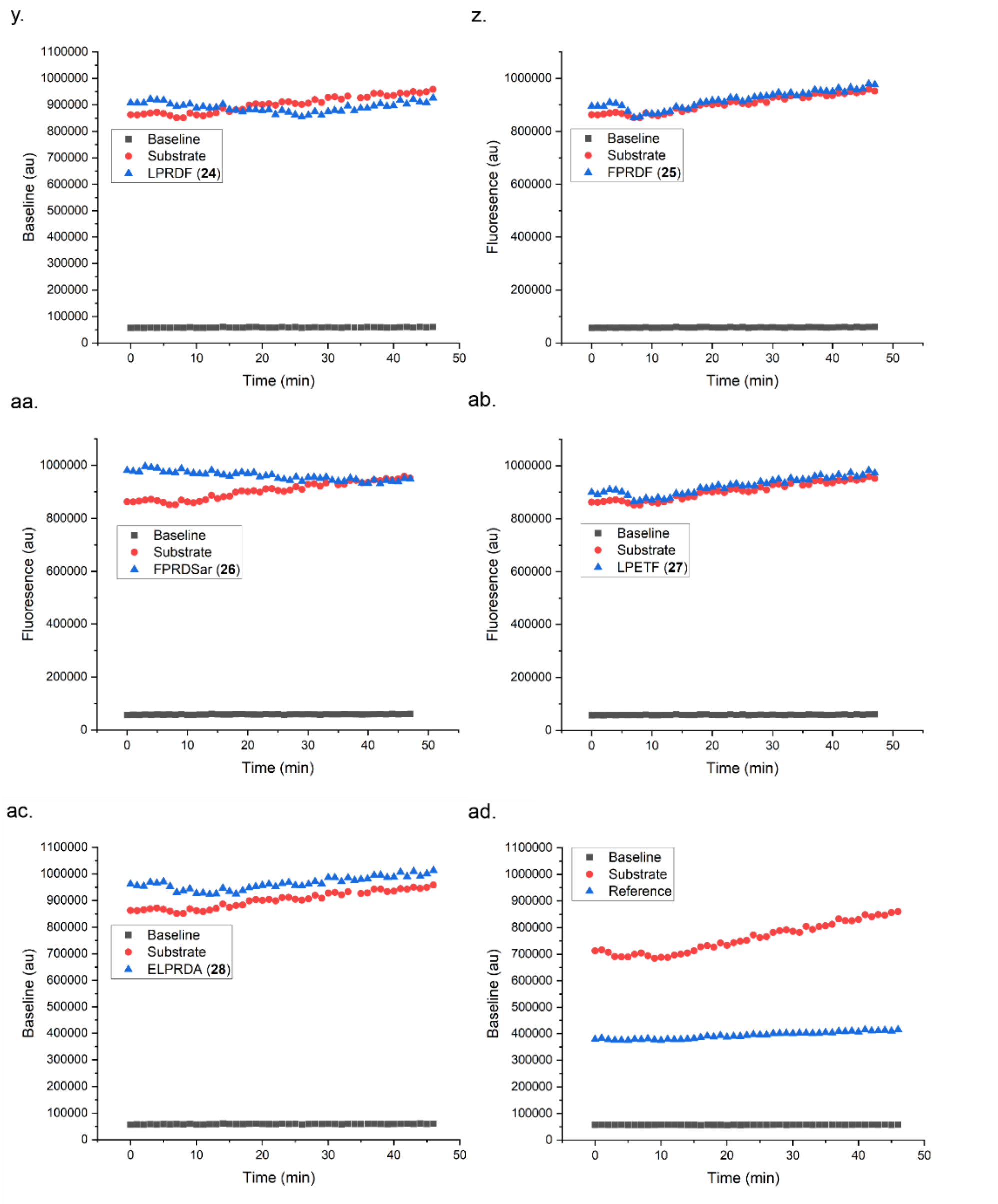

Figure S37. FRET-based assay results for the tested peptidomimetic compounds represented as fluorescence signal readouts plotted against time over 45 min for each compound. **A.** Abz-LPETGK(Dnp)-NH^2 Substrate, **b.** 1, **c.** 2, **d.** 3, **e.** 4, **f.** 5, **g.** 6, **h.** 7, **i.** 8, **j.** 9, **k.** 10, **l.** 11, **m.** 12, **n.** 13, **o.** 14, **p.** 15, **q.** 16, **r.** 17, **s.** 18, **t.** 19, **u.** 20, **v.** 21, **w.** 22, **x.** 23, **y.** 24, **z.** 25, **aa.** 26, **ab.** 27, **ac.** 28 and **ad.** reference. Each inhibitor was assayed in triplicate and average values were determined.

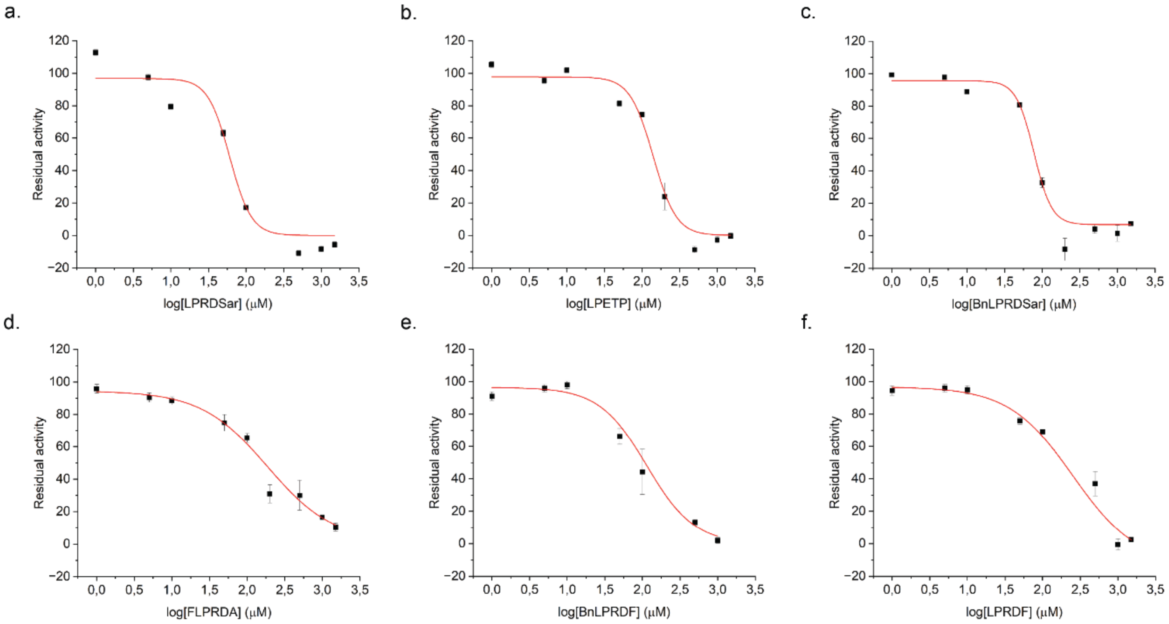

Figure S38. – Dose-reponse curves showing inhibition of SrtA activity in the presence of different peptides: **a. 5**; **b. 7**; **c. 18**; **d. 22**; **e. 23**; **f. 24**.

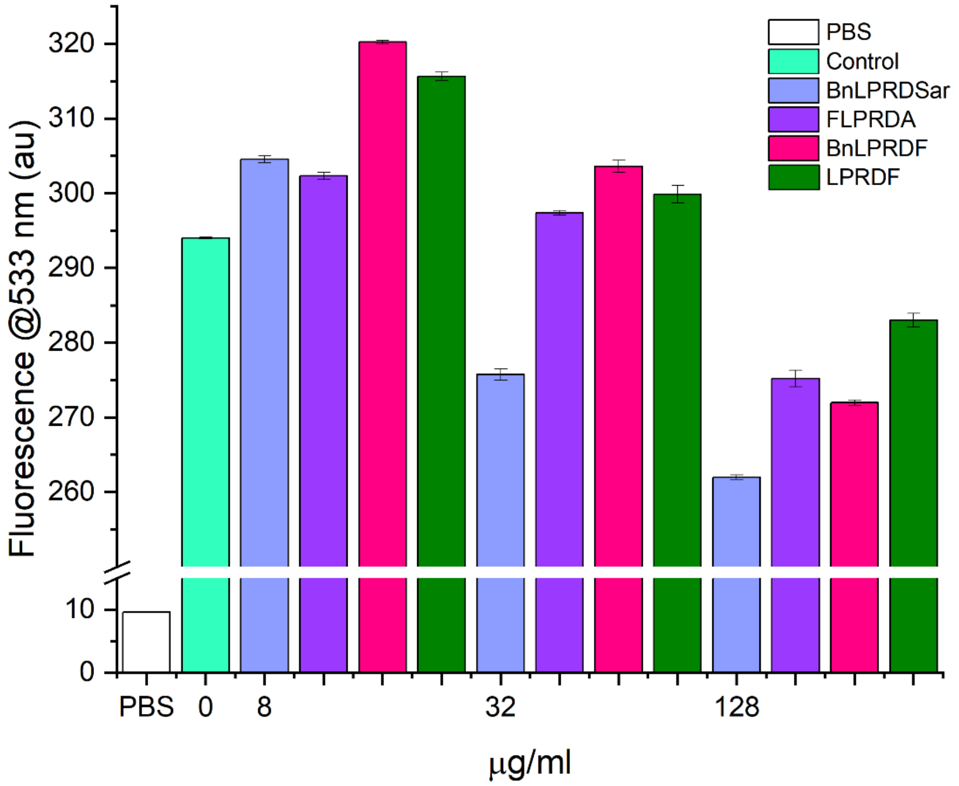

Figure S39. Fluorescence measurements of *S. aureus* stained with Sytox Green which were incubated with peptidomimetic inhibitors. Fluorescence measured at 533nm; samples were taken in triplicate, error bars are reported as standard error (±SE).

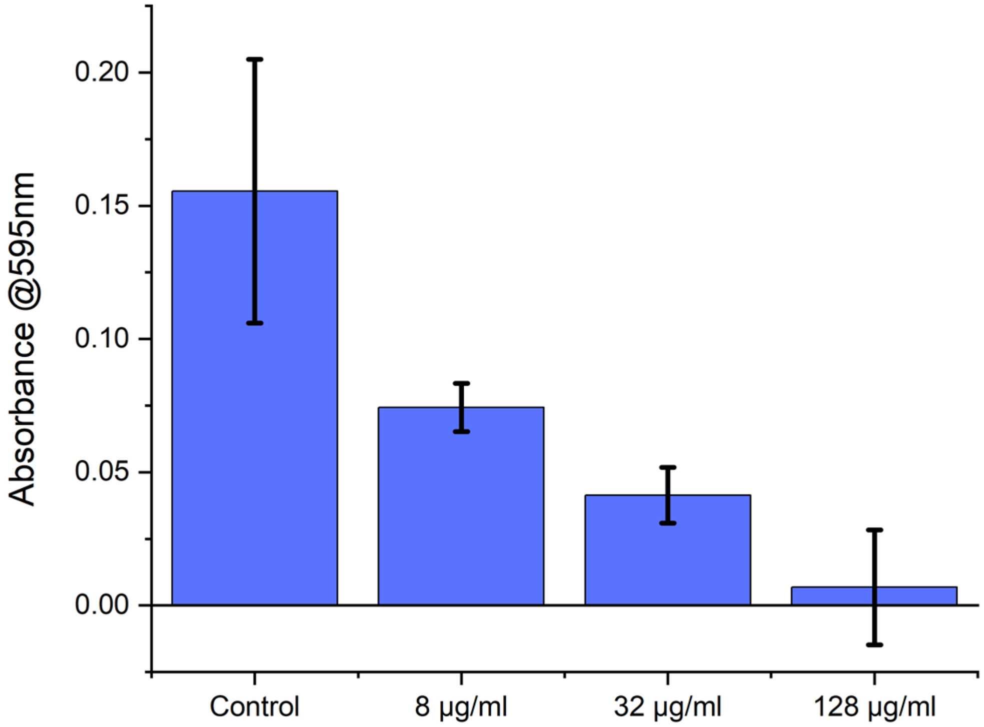

Figure S40. Absorbance measurements of *S. aureus* stained with Crystal Violet which were incubated with peptidomimetic inhibitors indicating biofilm inhibition. Absorbance measured at 595nm; samples were taken in triplicate, error bars reported as standard error (SE).

Table S2. Fluorescence values of *S. aureus* stained with Sytox Green and incubated with peptidomimetic inhibitors. Fluorescence measured at 533nm; samples were taken in triplicate.

	**PBS**	**Control**	**BzLPRDSar**	**FLPRDA**	**BzLPRDF**	**LPRDF**
**0 µg/ml**	9.63 ± 0.02	294.0 ± 0.1				
**8 µg/ml**			304.6 ± 0.5	302.3 ± 0.5	320.2 ± 0.2	315.7 ± 0.6
**32 µg/ml**			275.8 ± 0.8	297.4 ± 0.3	303.6 ± 0.8	299.9 ± 1.2
**128 µg/ml**			262.0 ± 0.3	275.2 ± 1.1	272.0 ± 0.4	283.0 ± 0.9

Computational Analysis

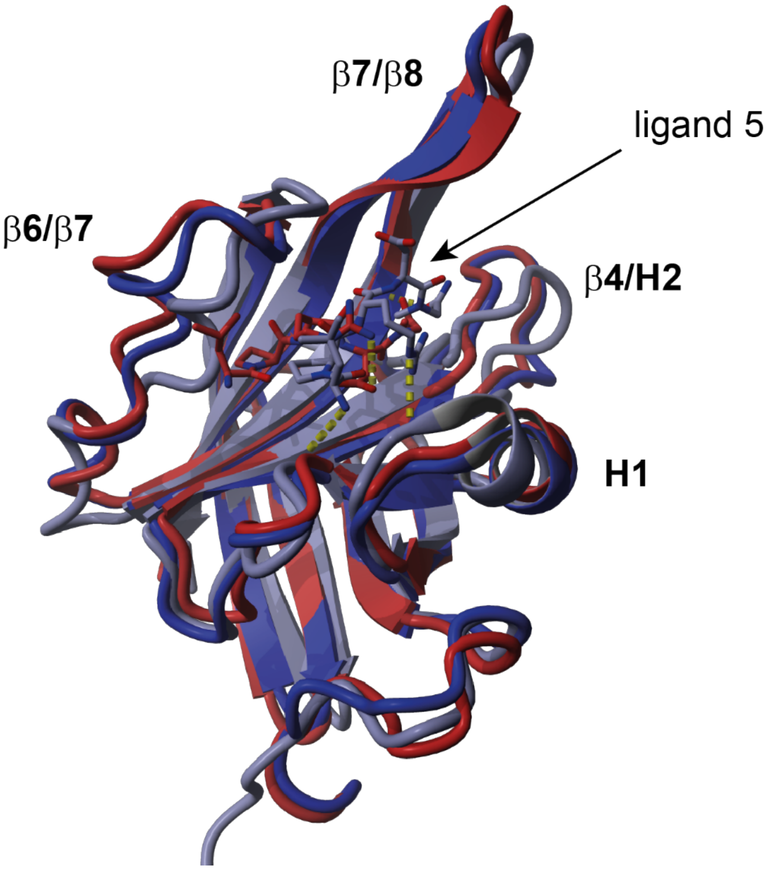

Figure S41. Optimized sortase A structures derived from pdb 2kid (SrtA*). Light-blue – energy minimized, initial SrtA structure from 2kid (lowest energy member from NMR ensemble) after ligand transfer form pdb 7S51 and transformation into ligand 5 (SrtA*), blue – SrtA* structure after 16 ns and 92 ns (red) of all-atom molecular dynamic simulation.

Table S3. Summary of docking results

	SrtA* structure	2kid-92ns				2kid-0ns			
#	ligand	Score [kcal/mol]		rank of selected docking conformer		Score [kcal/mol]		rank of selected docking conformer	
		Main	alternative	main/ alternative	hydrophobic interactions [kJ/mol]	main	alternative	main/ alternative	hydrophobic interactions [kJ/mol]
1	H_2N-LPRDA-OH	7.42		2	25.1	7.97		1	19.71
2	LPRDA-Ado[(a)]	7.03		1	31.7	6.70		2	30.21
3	Ado-LPRDA	7.49		1	36.33	7.26		1	33.66
4	LPRCA	6.60		4	29.85	7.12		1	28.64
5	LPRD-Sar[(b)]	6.78		1	29.65	6.80		1	14.80
6	LPET-Sar	7.25		1	27.57	7.60		1	36.21
7	LPETP	7.30		1	26.6	7.22	7.35	3/1 - inverse	23.40
8	KSFLPATGG AE	8.69		1	78.18	6.47		1	36.41

9	GTEPL	6.40		2	26.46	7.12		2	35.19
10	AcLPRDA	7.50		1	32.55	7.43		1	24.19
11	BzLPRDA	8.72		1	59.24	8.27		1	39.33
12	ChLPRDA	8.32		1	40.66	7.36		2	37.40
13	2NapLPRDA	9.10		1	53.22	9.07		1	86.36
14	CbzLPRDA	8.71		1	39.01	8.61		1	46.48
15	AzARDA[(c)]	7.01		3	16.63	7.60		1	23.27
16	LtARDA	7.95		1	30.42	6.97		1	35.37
17	LcARDA	7.14		3	30.94	6.50		2	21.12
18	BzLPRDSar	8.67		1	42.063	7.90	8.30	6/1 - inverse	43.63
19	BzLPETP	7.90		2	56.23	8.47		3	44.95
20	LPRDP	7.06		1	33.47	7.36		1	26.30
21	BzLPRDP	9.40		1	47.05	8.19		2	35.66
22	FLPRDA	8.29		1	48.104	8.87	9.13	3/1 - inverse	19.09
23	BzLPRDF	7.48	7.80	7/4	59.175	9.03	9.12	2/1 - inverse	50.33
24	LPRDF	6.68	6.83	4/2 - inverse	55.59	7.81	8.05	2/1 – inverse	43.65
25	FPRDF	8.00	8.30	4/1 - inverse	54.94	8.04		1	43.57
26	FPRDSar	7.93		1	40.33	7.27	7.56	4/1 - inverse	28.07
27	ELPRDA	9.39		1	34.92	6.64		2	34.50
28	LPETF	7.75	8.17	4/1 - inverse	37.69	8.43	8.75	2/1 - inverse	50.49

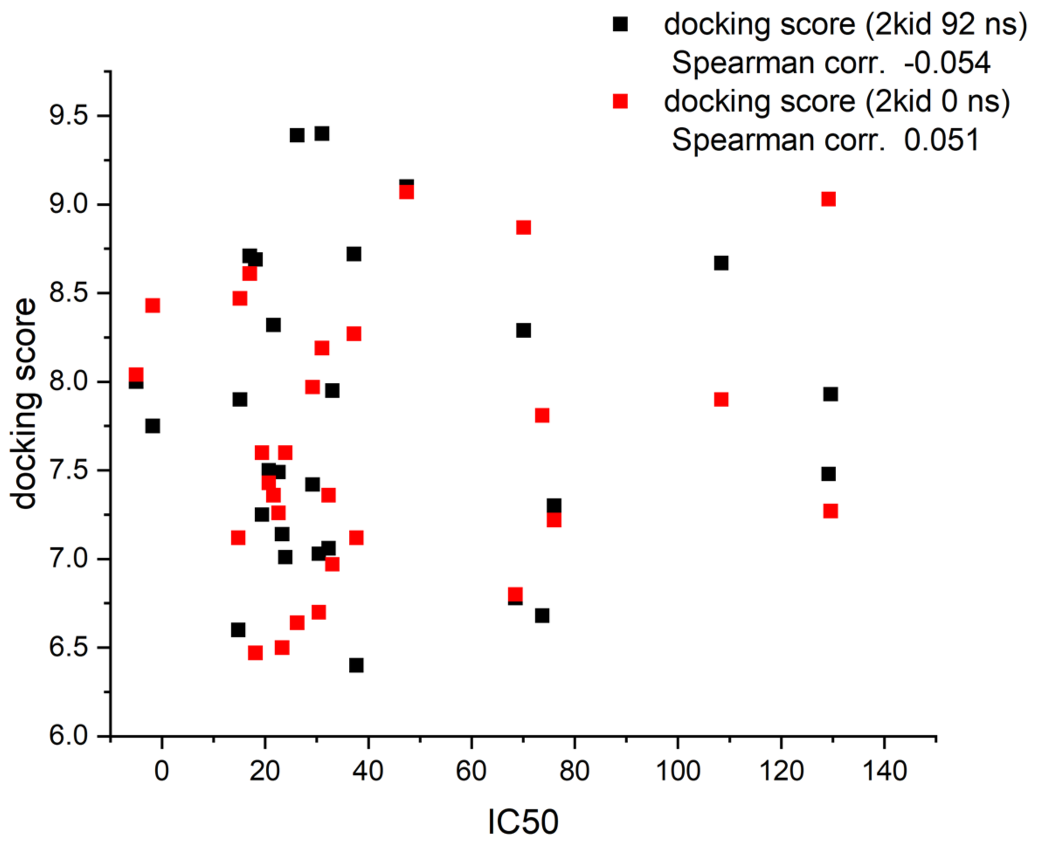

Figure S42. Correlation plot of the docking scores with the IC50 values. Spearman's rank order coefficients for both datasets are given in the figure legend.

References

1. D. Tietze, S. Voigt, D. Mollenhauer, M. Tischler, D. Imhof, T. Gutmann, L. González, O. Ohlenschläger, H. Breitzke and M. Görlach, *Angew. Chem. Int. Ed.*, 2011, **50**, 2946-2950.
2. H. D. Dickson, S. C. Smith and K. W. Hinkle, *Tetrahedron Lett.*, 2004, **45**, 5597-5599.
3. R. G. Kruger, P. Dostal and D. G. McCafferty, *Anal. Biochem.*, 2004, **326**, 42-48.
4. P. M. Wehrli, I. Uzelac, T. Olsson, T. Jacso, D. Tietze and J. Gottfries, *Bioorgan. Med. Chem.*, 2019, **27**, 115043.
5. S. Cascioferro, D. Raffa, B. Maggio, M. V. Raimondi, D. Schillaci and G. Daidone, *J. Med. Chem.*, 2015, **58**, 9108-9123.
6. E. Krieger and G. Vriend, *Bioinformatics*, 2014, **30**, 2981-2982.
7. E. Krieger and G. Vriend, *J. Comput. Chem.*, 2015, **36**, 996-1007.
8. O. Trott and A. J. Olson, *J. Comput. Chem.*, 2010, **31**, 455-461.
9. N. Suree, C. K. Liew, V. A. Villareal, W. Thieu, E. A. Fadeev, J. J. Clemens, M. E. Jung and R. T. Clubb, *J. Biol. Chem.*, 2009, **284**, 24465-24477.
10. D. A. Johnson, I. M. Piper, B. A. Vogel, S. N. Jackson, J. E. Svendsen, H. M. Kodama, D. E. Lee, K. M. Lindblom, J. McCarty and J. M. Antos, *J. Biol. Chem.*, 2022, **298**.
11. E. Krieger, K. Joo, J. Lee, J. Lee, S. Raman, J. Thompson, M. Tyka, D. Baker and K. Karplus, *Proteins*, 2009, **77**, 114-122.
12. U. Essmann, L. Perera, M. L. Berkowitz, T. Darden, H. Lee and L. G. Pedersen, *J. Chem. Phys.*, 1995, **103**, 8577-8593.
13. E. Krieger, J. E. Nielsen, C. A. Spronk and G. Vriend, *J. Mol. Graph. Model.*, 2006, **25**, 481-486.
14. E. Krieger, R. L. Dunbrack, Jr., R. W. Hooft and B. Krieger, *Methods Mol. Biol.*, 2012, **819**, 405-421.
